# The Tobacco Pack Surveillance System: A Protocol for Assessing Health Warning Compliance, Design Features, and Appeals of Tobacco Packs Sold in Low- and Middle-Income Countries

**DOI:** 10.2196/publichealth.4616

**Published:** 2015-08-12

**Authors:** Katherine Smith, Carmen Washington, Jennifer Brown, Alison Vadnais, Laura Kroart, Jacqueline Ferguson, Joanna Cohen

**Affiliations:** ^1^ Institute for Global Tobacco Control Department of Health, Behavior and Society Johns Hopkins Bloomberg School of Public Health Baltimore, MD United States

**Keywords:** tobacco products, cigarettes, public health surveillance, health communication, national health policy, marketing, developing countries

## Abstract

**Background:**

Tobacco remains the world’s leading preventable cause of death, with the majority of tobacco-caused deaths occurring in low- and middle-income countries. The first global health treaty, the Framework Convention on Tobacco Control (FCTC), outlines a set of policy initiatives that have been demonstrated as effective in reducing tobacco use. Article 11 of the FCTC focuses on using the tobacco package to communicate tobacco-caused harms; it also seeks to restrict the delivery of misleading information about the product on the pack.

**Objective:**

The objective of this study was to establish a surveillance system for tobacco packs in the 14 low- and middle-income countries with the greatest number of smokers. The Tobacco Pack Surveillance System (TPackSS) monitors whether required health warnings on tobacco packages are being implemented as intended, and identifies pack designs and appeals that might violate or detract from the communication of harm-related information and undermine the impact of a country’s tobacco packaging laws. The protocol outlined is intended to be applicable or adaptable for surveillance efforts in other countries.

**Methods:**

Tobacco packs were collected in 14 countries during 2013. The intention was, to the extent possible, to construct a census of “unique” pack presentations available for purchase in each country. The TPackSS team partnered with in-country field staff to implement a standardized protocol for acquiring packs from 36 diverse neighborhoods across three cities in each country. At the time of purchase, data on price and place of acquisition of each pack was recorded. The field staff, according to a standardized protocol, then photographed packs before they were shipped to the United States for coding and archiving.

**Results:**

Each pack was coded for compliance with the country-specific health warning label laws, as well as for key design features of the pack and appeals of the branding elements. The coding protocols were developed based upon prior research, expert opinion, and communication theories. Each pack was coded by two independent coders, with consistency of personnel across the project. We routinely measured intercoder reliability, and only retained variables for which a good level of reliability was achieved. Variables where reliability was too low were not included in final analyses, and any inconsistencies in coding were resolved on a daily basis.

**Conclusions:**

Across the 14 countries, the TPackSS team collected 3307 tobacco packs. We have established a publicly accessible, Internet archive of these packs that is intended for use by the tobacco control policy advocacy and research community.

## Introduction

### Policy Approaches to Tackling the Global Tobacco Epidemic

Tobacco use kills six million people each year and remains the leading preventable cause of death around the globe [1]. Almost 80% of tobacco-related deaths occur in low- and middle-income countries [2]. The World Health Organization’s Framework Convention on Tobacco Control (FCTC), the first global health treaty, outlines the evidence-based policies that countries should adopt in order to eliminate tobacco use [3]. The surveillance system outlined in this paper pertains to Article 11 of the FCTC, which requires that parties to the FCTC implement effective packaging and labeling measures to increase public awareness of the negative health impacts of tobacco products.

### The Cigarette Pack as a Powerful Communication Platform

The cigarette pack is on display not only when cigarettes are purchased, but also each time a smoker retrieves a cigarette from the pack [4]. A person who smokes a pack of cigarettes per day might look at a pack over 7000 times a year [5]. Moreover, it is not only smokers who are exposed to cigarette packs. Packs are in public view much of the time, either in the hands of smokers, left out at a social gathering, or prominently displayed in retail settings [5]. The cigarette pack has long been a key marketing method to attract new smokers and retain current ones [6-8]. As restrictions on tobacco marketing and advertising tighten, so tobacco packs have become an ever more important channel for brand advertising [6].

In addition to promoting smoking and prompting purchase of a specific brand, cigarette packs can also be used to provide important health information to smokers and the wider public. A growing number of countries are implementing health warning labels (HWLs) on cigarette packs, consistent with Article 11 of the FCTC [9]. As of September 2014, 77 countries or jurisdictions had adopted and finalized graphic HWL requirements [10]. HWLs have been associated with increased awareness of smoking risks; reduced appeal of smoking and smoking initiation among youth; increased motivation and intention to quit among smokers; increased cessation behaviors; increased use of cessation resources; increased likelihood that ex-smokers will remain abstinent; and reduced consumption levels among smokers [11-15]. Moreover, HWLs have been found to be a prominent and trusted source of health information for both smokers and nonsmokers [5,16]. Not all health warnings are, however, equally effective. Exposure to health warnings that are larger, placed on the front upper face of the tobacco package, and that contain pictures, are more effective than smaller text-only warnings [5,8,17,18]. While the impact of all warnings has been shown to lessen over time, pictorial warnings are less impacted by the “wear-out” effect [16,17]. Larger warnings allow for more information, such as cessation resources, to be provided to the consumer [15]. Graphic warnings that elicit a strong emotional response have been shown to be better remembered and reduce the urge to smoke [19].

In addition to adding warnings to packs, a number of countries now prohibit descriptors such as “light”, “mild”, and “low tar” on tobacco packaging on the basis that such terms can deceptively imply that these products are less harmful than other products. In the place of these descriptors, tobacco companies are using proxy terms (such as “smooth”), colors, and design elements to convey (falsely) that some products are less harmful than others [6,9,20-22].

### Study Overview

The Institute for Global Tobacco Control (IGTC) at the Johns Hopkins Bloomberg School of Public Health has developed the Tobacco Pack Surveillance System (TPackSS), to monitor whether required health warnings on tobacco packages are being implemented as intended, and to identify pack design features and appeals that might violate or detract from HWLs.

TPackSS was designed to be implemented in the 14 low- and middle-income countries with the greatest number of smokers [1]. The overall goal of the project is to collect one of every unique pack available for sale in each country. The initiative was developed with funding from Bloomberg Philanthropies through the Bloomberg Initiative to Reduce Tobacco Use [23].

## Methods

### Development of the Tobacco Pack Surveillance System Protocol

The development of the TPackSS protocol began with an exploratory and planning phase in 2012 to identify strategic goals and systematic protocols for establishing a surveillance system for cigarette packs. Based on discussions with 11 key tobacco control informants, a protocol was developed to collect packs as well as to train data collectors, photograph packs, code packs, and construct a searchable, Internet archive. Each expert consulted is affiliated with a leading academic institution or a public sector research or advocacy organization focusing on tobacco control issues. None of the consultants for this project have ties to the tobacco industry.

### Partnering With In-Country Data Collection Teams

In each country, IGTC collaborated with an in-country agency (market research firm; academic research group; nongovernmental organization, NGO; government-related agency; or independent consultant) to conduct field activities (see Table 1).

**Table 1:** In-country agency information and data collection dates.

Country	Agency type	Dates of collection
Bangladesh	NGO	September-October 2013
Brazil	NGO	January 2013
China	Academic research group	November-December 2013
Egypt	NGO	November-December 2013
India	Market research firm	October 2013
Indonesia	NGO	November 2013
Mexico	Government-related agency	July-August 2013
Pakistan	Independent consultant	November-December 2013
Philippines	Market research firm	April-May 2013
Russian Federation	NGO	September 2013
Thailand	Independent consultant	December 2013
Turkey	Academic research group	September-October 2013
Ukraine	Independent consultant	August 2013
Vietnam	Market research firm	June-July 2013

### In-Country Partner Selection

In-country partners were selected on the basis of having good English communication skills (oral and written), a strong background in research and field collection methods, working knowledge of the cities where data collection would take place, and the ability to define a sampling frame for neighborhoods and potential purchase venues in advance of data collection.

### Creation of the Sampling Framework for Each Country

The goal in constructing our sampling frame of tobacco vendors in each country was to maximize diversity in packs collected. The country’s most populated city and two additional cities of the next nine most populated cities were selected in each country based on cultural, geographic, religious, and linguistic diversity. An underlying philosophy of the Bloomberg Initiative is to focus on places with the greatest number of smokers, so as to maximize the potential impact of any given tobacco control intervention. Given this, we chose to focus data collection on diverse, populous cities within countries of interest. We recognize potential limitations in our sample’s focus on populous cities in terms of the possibility of excluding packs of products that are (almost) only consumed in rural areas.

**Table 2:** Data collection cities per country.

Country	City 1	City 2	City 3
Bangladesh	Dhaka	Sylhet	Chittagong
Brazil	São Paulo	Salvador	Manaus
China^a^	Beijing	Guangzhou	Shanghai
Egypt	Cairo	Alexandria	Mansoura
India	Mumbai	Delhi	Chennai
Indonesia	Jakarta	Semarang	Surabaya
Mexico	Mexico City	Guadalajara	Merida
Pakistan	Islamabad	Lahore	Karachi
Philippines	Manila	Cebu	Davao
Russian Federation	Moscow	Lahore	Karachi
Thailand	Chiang Mai	Bangkok	Hat Yai
Turkey	Istanbul	Diyarbakir	Konya
Ukraine	Kyiv	Lviv	Donetsk
Vietnam	Ho Chi Minh City	Hanoi	Da Nang

^a^In China, data collection was undertaken in 5 cities at the recommendation of tobacco control expert advisors. Additional cities were Kunming and Chengdu.

### Training In-Country Field Staff

One of two TPackSS staff traveled traveled to each country to train in-country field staff. Training was delivered in the initial city in which data collection took place in each country. For Egypt and Pakistan, social and political conditions at the time made travel from the United States inadvisable, and, therefore, in-country staff were trained in Dubai, United Arab Emirates. In each country, training took place over five days and included an overview of the project and its goals, hands-on instruction, and supervision with how to carry out data collection procedures, create a data inventory, and take standardized photographs of the tobacco packs collected. TPackSS staff accompanied in-country field staff for purchases in at least four neighborhoods in the initial city. In-country staff were also provided with detailed training reference documents (see Multimedia Appendix/ces 1-4) and remote access to staff for questions throughout the process of data collection, cataloging, and image creation.

### Pack Collection

Our aim in this project was to maximize breadth and collect one of every different/unique brand presentation available for sale in the tobacco vendors visited across 36 different neighborhoods in each country.

Within each city, 12 distinct neighborhoods were identified for tobacco vendor sampling. In-country field staff used a variety of local and national resources, including census and property value data, to create a sampling frame of low, moderate, and high socioeconomic areas within the metropolitan boundaries of each city. In each of three socioeconomic strata per city (high, medium, low), we selected four neighborhoods that were diverse in terms of geographic locale and residential composition. Thus, tobacco packs were acquired through purchases made at vendors from a total of 36 different neighborhoods in each country.

Unique packs were defined as tobacco packs that had at least one difference in an exterior feature of the pack. Any pack with a different design or feature, including packs differing in stick count, size, brand name presentation, colors, cellophane, and inclusion of a promotional item was considered to be “unique”. Packs that were exactly the same except for different iterations of the country’s warning labels were not considered to be distinct. Although the majority of packs were easily identified as unique (ie, Marlboro Red was identified as distinct from Marlboro Blue), some packs had minor differences that were more difficult to discern, such as differences in cellophane wrapping around the pack or variations in smaller pack features like brand logo.

In addition to cigarette packs, we also collected cigarros de palha (straw cigarettes from Brazil) and packs sold with promotional items, where these were sold alongside cigarettes. Promotional items were defined to be products that contained a tobacco pack and an additional item such as a lighter, ashtray, or hard tobacco pack carrying case. In countries where bidis or kreteks were sold, these were also purchased.

The initial (index) purchase was always made from a large tobacco vendor in the first sample city, where a broad array of tobacco products was available. We expected the index store purchase to be the largest purchase in each country. In-country staff identified the initial/index vendor in advance of the first day of data collection.

At the initial purchase, every distinct pack available for purchase was acquired, and the price was recorded for each item purchased. The purchase of packs required two field staff. One field staff member worked systematically to review and select every unique cigarette pack on display. The other staff member recorded price and organized purchased packs so that information on pricing of each pack was retained. After identifying and purchasing one of each distinct pack that was visible, field staff asked the vendor whether there were any additional packs for sale that were not visible, and if available, these packs were also purchased. Where itemized receipts were not provided, staff hand-recorded pack identification numbers (IDs) and price paid before leaving the place of purchase/store. Where permissible, staff took a photo of the tobacco pack display from which packs were identified and purchased. After the purchase, staff used a tablet-based data entry system using the application doForms [24] to record descriptive details about the purchase including city, socioeconomic neighborhood, number of packs purchased, type of vendor, and date of purchase (see Multimedia Appendix 2). Forms were completed and saved and uploaded once Wi-Fi became available.

After the purchase in the initial store, the team returned to the field office where each pack was placed in an individual bag and labeled with a unique ID convention that identified for each pack the country, city, socioeconomic status of the neighborhood, and an assigned pack ID number (see Multimedia Appendix 2). The pack price, type of vendor, and the brand name were also added to the label. Photographs were then taken of the front panel of each pack alongside the ID label and uploaded to a tablet computer to create an archive of purchased packs. Brand families were identified by in-country field staff and were defined as a group of brands (ie, Marlboro Red, Marlboro Blue) that are related and have a parent brand (ie, Marlboro). Brand family folders were created on the tablet, and each purchased pack’s front panel image was added to its designated brand family folder. Any pack whose image was in the tablet reference archive was not purchased from subsequent vendors visited. For each country, packs were verified for duplicates upon receipt of the physical packs in Baltimore, Maryland.

The two field staff then visited the 35 remaining neighborhoods (across the 3 cities), and in each neighborhood one vendor was purposively selected based on having a large product inventory. Using the tablet, field staff systematically reviewed all packs available and purchased any pack that did not already appear in the image archive. In the event that the identified vendor did not have any new packs, field staff visited up to three more vendors in the same neighborhood, and made a purchase from the first of these vendors to have any new packs, before moving to the next neighborhood.

### Data Inventory Creation

Research Electronic Data Capture (REDCap) [25] was used to manage inventory data. REDCap provides an interface for validated data entry and creates audit trails for tracking data manipulation. In-country field staff undertook initial entry of data on the packs purchased as soon as possible after the purchase was made. IGTC staff checked REDCap data entry regularly during data collection. The data entered on each pack were: pack ID, brand name (Roman as well as any linguistic characters), price, date of purchase, type of tobacco product, manufacturer as presented on the pack, and place of manufacture as presented on the pack. Data access groups were created for each in-country team and user rights were revoked at the completion of data entry to prevent accidental data deletion and to reduce data errors (see Multimedia Appendix 3).

### Photographing the Packs

In-country staff created pack images by using a detailed protocol (see Multimedia Appendix 4). Field staff were provided with all photography equipment, including camera, tripod, and lighting equipment. Training on the photography process was covered in one day, with daily practice and review by TPackSS staff during the first week of data collection.

In-country staff took nine standard images of each pack: one direct image of the front panel; one 45 degree angle photo of the front and side panel; one each of the back, top, bottom, and both side panels; one of the opened pack; and one of the cigarette stick. In addition to these nine standard images, in-country staff also captured any other text, including branding on the cellophane and branding revealed when a pack is opened (eg, under the lid). The photos were organized into individual folders by their respective unique identifiers and folders were uploaded to a cloud-based storage application, where they were downloaded by TPackSS staff in Baltimore and checked for image quality. Any images that did not meet protocol standards (eg, improper lighting, unclear text) were identified by TPackSS staff and were retaken by in-country field staff. After initial quality control, TPackSS staff in Baltimore edited each image individually for sizing and brightness necessary for upload to the TPackSS website.

### Shipping and Receiving the Packs

All packs were shipped to Baltimore from the country of purchase to facilitate coding for warning label compliance, design features, and pack appeals. Approval was obtained from the US Food and Drug Administration and the Federal Trade Commission to import and store packs for research purposes. Packs were (and continue to be) stored in the TPackSS offices in labeled and catalogued boxes in locked filing cabinets.

As shipments of packs arrived at the TPackSS office in Baltimore, information on the packs was added to a spreadsheet on Google Drive that included the pack unique ID, information about arrival and storage, price paid for the pack, date purchased, vendor of purchase, brand name, brand name in Roman characters (where the initial name was not in Roman characters), tax stamp presence, product type, health warning rotation, verification that the physical pack matched the website pictures, and faces of the pack that had text where translation was necessary. The original inventory data captured by in-country field staff was verified through this process. Missing and duplicate packs were also identified. All packs within a country were systematically checked for the presence of duplicates by comparing each pack to every pack in its respective country.

In the few instances of missing packs upon arrival in Baltimore, staff followed up with in-country partner agencies, and if possible, duplicates of packs missing from the physical dataset were purchased and sent to TPackSS researchers. There were a total of 17 missing packs from the collection upon receipt of shipment (10 from India, 3 from Russia, 1 from Turkey, and 3 from the Philippines). We received 12 replacement packs in total (8 from India, 3 from the Philippines, and 1 from Turkey), with 5 packs remaining missing (2 from India and 3 from Russia). Of the 5 remaining missing packs, US Customs held 2 because they were manufactured in Cuba.

Using Google Drive allowed for data entry by multiple people simultaneously, and saving a copy of the spreadsheet at the end of each day new data were entered ensured quality. Packs were sorted and stored alphanumerically and by presence of specific health warnings; all packs with a given health warning were stored together for the ease of coding health warning compliance. Packs were resorted by brand name once health warning compliance coding was completed.

### Translation

We employed a professional translation service to translate non-English text on the packs. In most cases, it was not necessary to translate warning label text because warning label text on packs could be directly compared with copies of approved warning labels from each country. The translation service was provided with the number of the image as found on the TPackSS website for each pack panel where any translation was required. All translation was entered into a database with rows for each pack and columns ordered by panel. The translation service provided transcription of the text, literal translation, and adaptive translation for cultural meaning when applicable. The translation database was used during coding for compliance with HWL laws and features and appeals (see below).

### Creating Codebooks for Pack Compliance With Health Warning Labels Laws

We created a codebook for each country based on the tobacco packaging and labeling laws in effect in each country at the time of data collection. All laws were acquired from the Tobacco Control Laws website [26], a public resource maintained by the Campaign for Tobacco-Free Kids. For countries whose laws were not written in English, we utilized the unofficial English translation of the law, provided on the Tobacco Control Laws website. Only those requirements concerning labeling and packaging were incorporated into the codebooks. Where applicable, the codebooks included measures of inclusion of information on HWLs, emissions and content levels, indications of less harm such as misleading descriptors, and messages prohibiting the sale of cigarettes to minors (see Multimedia Appendix 5).

Any aspect of a law that was considered too nonspecific to be coded consistently was excluded from the codebook. Legal and country experts were consulted when interpretation of the law was in question. Each codebook went through multiple iterations to improve the validity and the reliability of the coding process. During the codebook development process, two members of the research team coded packs in order to assess the reliability of the variables at each stage. Where differences in coding interpretation were identified, codebook questions were clarified. The research team also considered the codebook variables in light of available packs from each country to anticipate coding challenges not obvious from laws alone. Challenges were recorded, discussed, and resolved by the research team.

### Creating a Codebook for Features and Appeals

In addition to compliance with tobacco packaging and labeling laws, we also coded each pack for its physical, textual, and visual aspects (features and appeals). Unlike country-specific codebooks to assess compliance with HWL laws, there is one common “Features and Appeals” (F&A) codebook used for all countries (see Multimedia Appendix 6). In order to develop the codebook for F&A, we reviewed the tobacco control literature on packaging and marketing [6,7,27-29]. We also consulted existing coding systems for tobacco packaging F&A, such as the Chatterbox website [30]. We sought to integrate relevant concepts from the published literature on brand appeal, market development, and audience segmentation into our F&A codebook.

**Figure 1:**
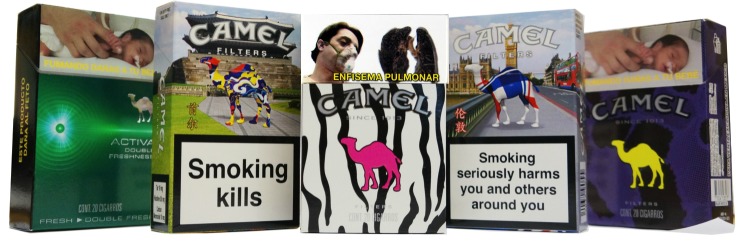
Illustrative photo of collection packs.

Each pack was coded for “features”, which pertain to design elements of the pack including the shape and size of the pack, color, size descriptors, Web presence, the type of opening, and any wrapping or container. The outside and the inside of the pack were both considered, as was the product (stick) itself. We also coded for any evidence of various common “appeals” associated with the tobacco products. Product “appeals” are connections and connotations created in marketing efforts in order to create reasons for a person to purchase (or desire to purchase) a given item (Figure 1 shows this). The TPackSS “appeals” codes are assessments of sociocultural connotations made via various visual elements of branding on the pack to create positive sentiments about the product among a target audience [30]. Product “appeals” can appear both on and inside the pack, on any wrapping or additional packaging, as well as on the stick. We looked for both lexical (words) and images that convey specific appeals. Our appeals codes included (but were not limited to): technology, luxury, femininity, masculinity, youth, nationalism, and United States.

An initial draft of the codebook was developed and then refined to improve objectivity and reliability of coding categories. The coding development and refinement involved test coding sample packs from all 14 countries and a wide variety of pack shapes and opening styles. In addition, we consulted in-country professionals with expertise in tobacco control, communications, and marketing, on the interpretation of culturally significant imagery, and how to objectively code for elements of nationalism and cultural appeal.

### Coding the Packs Overview

Packs were first coded for compliance with HWL requirements. Only packs displaying a HWL that had been issued by the country in which the pack was purchased and that was in rotation at the time that packs were collected were coded for HWL compliance.

A subsequent and separate process was undertaken for coding packs for F&A. All packs collected (regardless of existence of appropriate HWL) were coded for F&A. In each instance, two independent coders coded every pack. The coders all went through extensive training in tobacco control policy and packaging features, and to the extent possible, the coders were involved in the development of the various codebooks.

TPackSS staff completed all coding using the physical packs rather than the images. To retain a high level of coding quality, coding was limited to approximately 4 hours per day. At the beginning of coding for any country, a team meeting was held to present and discuss the HWL compliance codebook with coders. When necessary, codebooks were revised for clarity and coding issues were resolved.

Over time, one pair of coders specialized in HWL compliance coding, and two sets of coders specialized in F&A coding. All differences or discrepancies within the coding pairs were discussed in regularly held review meetings so as to resolve differences and refine and improve the coding processes. All coding discrepancies and subsequent resolutions were recorded and stored in a central repository. REDCap was used for data entry [25].

In addition to individual pack coding, we also undertook summative and comparative consideration of groups of packs. For HWL compliance, we compared all packs to be coded within each country by warning label and looked for differences in size, color, warning image distortion (such as being stretched or only showing part of the image), placement on the pack, and initial consideration of content. For F&A, we gathered brands across countries and considered all elements of brand consistency and the impact of health warning placement on brand display. In each instance, this was undertaken as a group process with notes taken about notable elements and possible patterns throughout the sets of packs being considered.

### Data Management and Quality Control

Database management began with raw data imported from doForms and REDCap and ended with a frozen analytic file with a corresponding codebook. All data were stored in Stata 13 in 2013 to 2014 [31] and then Stata 14 [32] in 2015. Data were accessible only to the database administrator and the database administrator was accountable for all corrections, addition, deletions, and merging of data. Data were routinely backed up on an encrypted external hard drive.

Data audit trails were produced for every addition, deletion, and change of the original data. Multiple data validation checks (eg, extraneous values, outliers, and abnormalities), data verification within a dataset (eg, simple range and constraint validation), and cross-dataset checks (eg, data redundancy and consistency checks) were performed.

For each country, there are five initial datasets that were merged after data cleaning to form a master relational dataset. In order of their generation, the datasets are: (1) Field data; (2) Intake data; (3) HWL compliance; (4) Brand Names and Owners; and (5) F&A. Each pack’s unique identifier enabled the merging of different datasets into one master relational database. Field data were verified and cross checked against Intake data. Intake data were verified and cross checked against HWL compliance coding, and so forth. Consulting tobacco brand and tobacco brand owner websites validated brand names. Consulting Euromonitor International [33], a market intelligence report based upon tobacco market research, also validated brand names. For the purpose of this study, "Owner of the Brand" is defined as the entity that holds an active trademark registration of the brand in the particular country and/or would be responsible for the pack in instances of any legal or business related challenges, absent other information. A consultant who utilized portfolios of brands and trademark registry databases validated brand owners.

Because the F&A and HWL compliance data were both entered by independent coding pairs, any discrepancies were reconciled through a process of review by a third trained reviewer. All reconciliations were performed within 24 hours of entering the data for F&A coding and every other day for HWL compliance coding.

Pack measurements (ie, pack height and warning label height) in the HWL compliance codebook required special attention. Coders measured packs with standardized rules and rounded measurements to the closest millimeter (mm). Given that it was infrequent, although not rare, to get exactly the same measurement between coders, a difference of 1 mm was averaged, while a difference greater than 1 mm required measurement by a third reviewer. Bland-Altman plots were created to assess random error and systematic error in the coder’s measurements [34]. Quality checks were also performed on the HWL compliance data to ensure impossible data were not being entered (eg, width of the warning label greater than the width of the pack).

To assess data reliability in the F&A and HWL compliance codebook, Cohen’s kappa, prevalence adjusted kappa (PABAK), and interclass correlation coefficient were calculated as appropriate for each country [35-39]. Each coder’s entry for a record was compared against the final merged record to assess how frequently coders were agreeing with the final record. The PABAK statistic takes into account low-prevalence bias that skews the Cohen’s kappa. By evaluating both of these statistics, we identified variables with low agreement (< 80% agreement). A 2-sided alpha of < .05 was considered statistically significant. Variables with low agreement were flagged and coders were routinely provided feedback on variables for which there was low agreement. This feedback was used to clarify instructions for the codebook. Follow-up was performed to ensure agreement of these variables improved as coding progressed.

### Developing a Health Warning Label Compliance Score

Article 11 of the FCTC outlines mechanisms by which parties to the treaty can increase the effectiveness of their tobacco packaging and labeling. Key elements include location; size; use of pictorials; color; rotation; message content; language; source attribution; and information on constituents and emissions.

We operationalized HWL compliance through four overarching categories that related to requirements across the study countries: (1) Warning location; (2) Warning size; (3) Text size in the warning; and (4) Warning label elements (such as color or content of warning). All four key requirements were assessed for 10 countries. For the four remaining countries, only 3 of the 4 categories pertained to the country’s law, and so only these elements were included in the compliance measure (Table 3). The compliance score was calculated as number of compliant packs divided by number of packs with a warning label in rotation at the time of collection. In addition, a composite compliance score was determined by dividing the number of packs compliant on all four key requirements by the total number of packs coded for each country. The compliance results are outside of the scope of this protocol paper.

**Table 3:** HWL compliance measures as related to elements of countries’ laws.

Country	Warning type & location	Warning size	Warning text size	Warning label elements
Bangladesh	Text warnings on front and back of the pack	30% of the front and back	18 point font	Black text on white background or white text on black background
Brazil	Picture-based warning on back of the pack	100% of the back	Proportion and graphic parameters of images provided by the Brazilian Health Surveillance Agency must be unchanged	White text on black rectangular background
China	Text warnings on front and back of the pack	30% of the front and back	4 mm tall text	Color contrast between the text and background
Egypt	Picture-based warning on front and back of the pack	50% of the front and back	Not applicable	Text printed on black background; quit line and standard warning printed on yellow background
India	Picture-based warning on front of the pack	40% of the front	Warning must be 0.75 to 1 ratio of its vertical to horizontal length	Red and white text on black background
Indonesia	Text warnings on a “part of the package that is easily read”	Not applicable	3 mm tall text	Black text on background that is a shade of white with black border
Mexico	Picture-based warning on front and text warning on back of the pack	30% of the front and 100% of the back	10 point font on front; 9-11 point font on back	Yellow text on front and back; black background on back
Pakistan	Picture-based warnings on front and back of the pack	40% of the front and back	2 mm tall text	Black text on white background
Philippines	Text warnings on front of the pack	30% of the front	Text must comprise at least 50% of the warning	Black text on white background with black border
Russian Federation	Text warning on front and picture-based warning on back of the pack	30% of the front and 50% of the back	Not applicable	Black border on front and back; black text on white background on back
Thailand	Picture-based warnings on front and back of the pack	55% of the front and back	Size and positioning of text must appear as it does in examples provided by the Ministry of Health	Content must appear as it does in examples provided by the Ministry of Health
Turkey	Picture-based warning on front and picture-based warning on back of the pack	65% of the front and back	Not applicable	Black border on front and back; black text on back
Ukraine	Text warning on front and picture-based warning on back of the pack	50% of the front and back	Must occupy no less than 40% of the area within the black border of the health warning	Black text on white background with black border
Vietnam	Text warnings on front and back of the pack	30% of the front and back	2 mm tall text	Black text on white background

## Results

### Packs Collected

Across the 14 countries, we collected 3307 tobacco packs. We collected 3006 cigarette packs, 55 bidi packs, 234 kretek packs, 3 cigarros de palha (straw cigarettes), and 9 promotional items. Product type was determined by labeling on the pack. We collected the most packs from the Russian Federation (n=505) and the fewest packs from Egypt (n=58). We will be analyzing data on compliance with HWL requirements, pack F&A, and pricing of products in separate analyses to be published at a later date.

**Table 4:** Packs collected in each country and each city.

Country		Number of packs collected		
	Total	City 1	City 2	City 3
Bangladesh	200	143	11	46
Brazil	130	104	10	16
China^a^	453	227	70	55
Egypt	58	58	-	-
India	169	108	29	32
Indonesia	215	115	35	65
Mexico	134	107	19	8
Pakistan	394	296	58	40
Philippines	144	79	53	12
Russian Federation	505	406	61	38
Thailand	126	57	53	16
Turkey	308	206	72	30
Ukraine	324	242	48	34
Vietnam	147	120	24	3
Total	3307^a^	2268	543	395

^a^Total includes packs from China city 4=50 & city 5=51 packs

### Creating a Website

**Figure 2:**
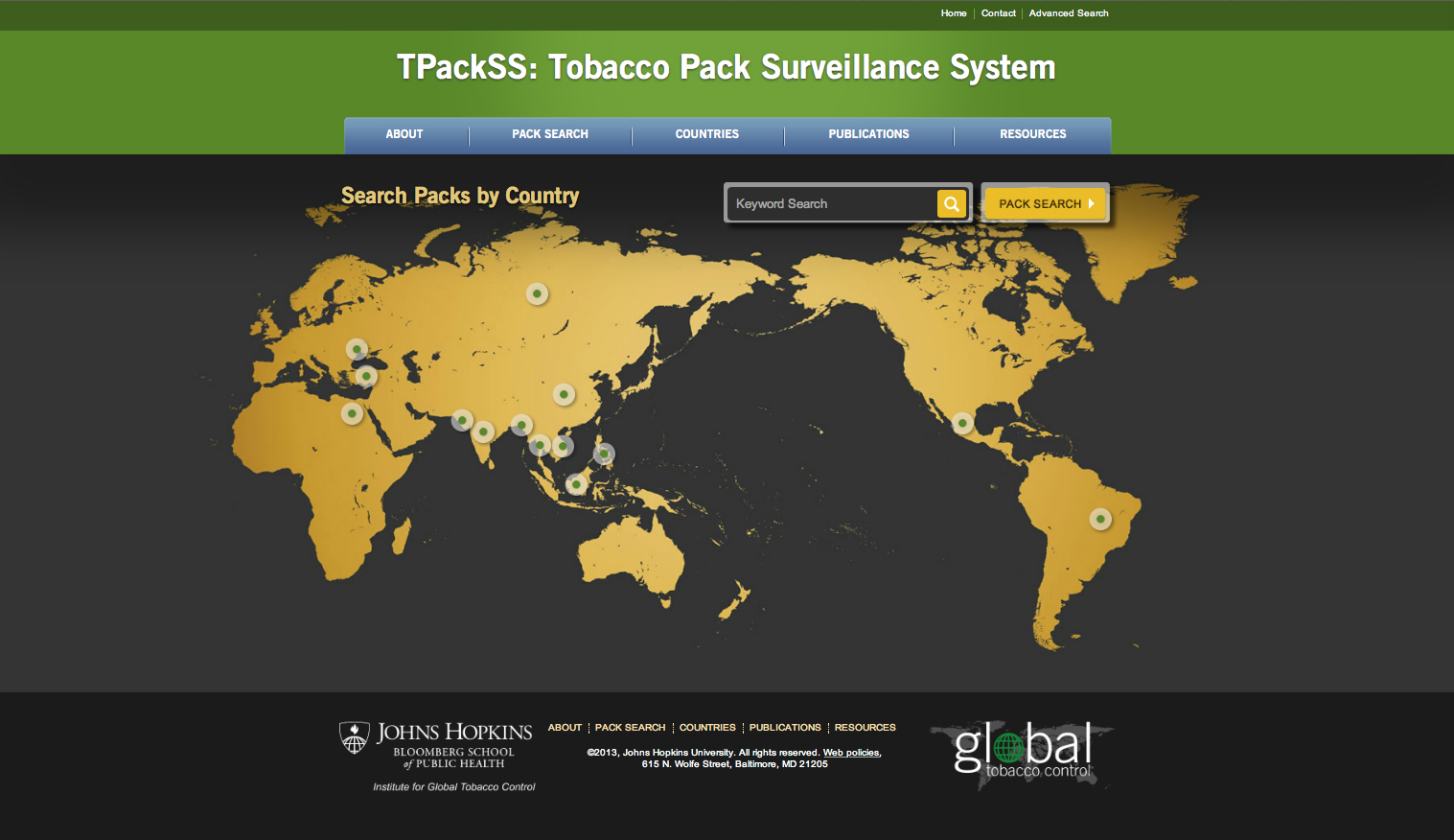
Screenshot of Internet archive.

The TPackSS searchable Internet archive is now publicly available [40] (Figure 2 shows this). This dynamic archive houses images of all the tobacco packs purchased in the 14 countries. The intention is for this archive to be used by the tobacco control community to monitor compliance with existing HWL laws, understand innovation in pack design and brand promotion, and advocate for policy change that can prevent future harm from tobacco use.

A Web development company was contracted to create a site that can be searched and filtered by country, brand family, brand owner, and tobacco product type. Pack specific data such as brand family, product type, price, purchase date, purchase city, and purchase country were paired with each pack and are also available on the site. The site includes information about packaging and labeling regulations in each country.

Future plans for the website include uploading the key compliance, design, and F&A variables for each pack, and incorporating the capacity to view the site in multiple languages.

## Discussion

TPackSS data collection will be repeated at a minimum of two years following the previous data collection, for those countries where the warning label or packaging requirements have changed. This will facilitate comparisons of packaging pre and post policy implementation.

In addition to creating the Internet archive, we are compiling country-specific fact sheets on HWL compliance to support policy advocacy. We are also creating resources (country specific and comparative) related to various aspects of the F&A. The fact sheets will be downloadable from the TPackSS website [40]. We have made our field protocol documents, health warning compliance codebooks, and F&A codebook available on the project website.

## Appendix

Multimedia Appendix 1 Training manual.

Multimedia Appendix 2 In-field data collection form for vendor and neighborhood.

Multimedia Appendix 3 REDCap in-country inventory form.

Multimedia Appendix 4 Photography protocol.

Multimedia Appendix 5 Example of one country health warning compliance codebook.

Multimedia Appendix 6 Features and appeals codebook.

## Abbreviations

F&A: features and appeals
FCTC: Framework Convention on Tobacco Control
IGTC: Global Tobacco Control
HWLs: health warning labels
IDs: identification numbers
mm: millimeter
NGO: nongovernmental organization
PABAK: prevalence adjusted kappa
REDCap: Research Electronic Data Capture
TPackSS: Tobacco Pack Surveillance System
