# Supplementary material for: The Tobacco Pack Surveillance System: A Protocol for Assessing Health Warning Compliance, Design Features, and Appeals of Tobacco Packs Sold in Low- and Middle-Income Countries
Source: JMIR Public Health Surveill. 2015 Aug 12;1(2):e8. doi: 10.2196/publichealth.4616 (PMC4869212; doi:10.2196/publichealth.4616)
Supplement: Multimedia Appendix 1 [file publichealth_v1i2e8_app1.pdf]

AGENDA: TRAINING AND DATA COLLECTION

City 1

Day 1: Introductions and Training

Morning: IGTC and project overview; review of field collection protocol; discussion about neighborhoods and vendors; overview of in-country activities

Afternoon Activities: Training on doForms application; training on REDCap database; training on unique ID structure and archiving images in iPad

Day 2: Training and Field Collection

Data collection at first site; label packs; archive images in iPad; practice using REDCap

Day 3: Training and Field Collection

Training on inventory and image archiving; data collection

Day 4: Training and Field Collection

Training on packaging and shipping; data collection

Day 5: Training and Field Collection

Review and practice protocol for archiving, saving, & uploading images; review plans for City 2; Data collection

City 2

Day 6: Travel to City 2; debrief field work; review and plan data collection activities in City 2

Day 7: Data collection

Day 8: Data collection; debrief field work

City 3

Day 9: Travel to City 3; review and plan for collection in City 3

Day 10: Data collection

Day 11: Data collection; debrief field work

Day 12: Travel back to City 1

BACKGROUND

Johns Hopkins Bloomberg School of Public Health, Institute for Global Tobacco Control (IGTC) is monitoring whether required health warnings on tobacco products are being implemented as intended, and identifying pack design appeals that might violate or detract from country tobacco packaging laws through the development of a surveillance system of tobacco product packaging in the 14 Bloomberg Initiative priority countries.

Study Design

Cities

In each country, data collection occurs in a sample of three cities that includes the largest populated city in the country and two additional cities. The two additional cities are chosen from the top 10 populated cities on the basis of cultural, geographic and potentially linguistic diversity. Data collection begins in the largest city, and then proceeds to the next two cities. The order of data collection in cities 2 and 3 is determined largely by pragmatic factors (distance, cost, and travel logistics).

Neighborhoods

In each city we identify low, middle and high socioeconomic neighborhoods, and then target **four** characteristically distinctive neighborhoods within each of these three strata. The distinct neighborhoods can be based on geographic location the makeup of who lives in the neighborhood (e.g. immigrants, religious group, ethnic group, professionals) or landmarks (e.g. University area, tourist area) for each socioeconomic area. For each city, a total of **12** distinctive neighborhoods are identified.

Vendors

Within each distinctive neighborhood one vendor is identified for the purchase of cigarette packs (additionally, cigarillos, packs sold with promotional items, where these were sold alongside cigarettes, and in countries where bidis or kreteks were sold, these were also purchased). GATS country level data is assessed in advance to identify the type of vendors where consumers purchase tobacco packs. Within each neighborhood the GATS-identified types of vendors are purposively selected based on the size of their tobacco product inventory. Vendors should represent retail outlets where a large variety of cigarette brands are sold.

In the first city an “initial major vendor” is identified, where a large, initial purchase takes place. At this vendor one pack of every unique pack design available is purchased. At the 35 remaining vendors, one of every unique pack design that has not already been purchased at a previous vendor is purchased.

Twelve vendors are visited in each city: one vendor in each of **four distinct neighborhoods** in each of the low, medium, and high socioeconomic areas. Our total sample consists of packs collected from a total of **36** vendors in each of the three cities (see Vendor Matrix below).

	Low	Middle	High	# vendors (Total = 36)
City 1	4 distinct neighborhoods<br>**= (4 vendors)**	4 distinct neighborhoods<br>**= (4 vendors)**	4 distinct neighborhoods<br>**= (4 vendors)**	12
City 2	4 distinct neighborhoods<br>**= (4 vendors)**	4 distinct neighborhoods<br>**= (4 vendors)**	4 distinct neighborhoods<br>**= (4 vendors)**	12
City 3	4 distinct neighborhoods<br>**= (4 vendors)**	4 distinct neighborhoods<br>**= (4 vendors)**	4 distinct neighborhoods<br>**= (4 vendors)**	12

Special note: If the selected vendor in a specific neighborhood does not have any new packs (packs which were not previously purchased at another store), then up to three additional vendors are visited in this neighborhood until a new pack(s) is found.

DATA COLLECTION

Initial Major Vendor Collection (time in store approx. 30 minutes to 1 hour)

Objective: Acquire one pack of every unique cigarette pack design (all cigarette brands and their variants and multiple brand designs) available at this first site. Before the visit, staff contacts the store's management to confirm a convenient time or schedule an appointment for the purchase of the products.

Team member roles (2 field workers required)
Person 1: primarily interacts with the vendor; is in charge of systematically identifying the cigarettes for purchase; is in charge of paying for the purchase and acquiring the receipt for purchase.

Person 2: is primarily responsible for management of the packs, including audio recording identifiable information about the pack, packing and labeling each individual pack, and recording the price paid for the product.

Equipment:	☐ Sony camera
☐ Sony audio recorder	☐ writing pens
☐ Ziploc sandwich bags	☐ carry bag
☐ ID labels	☐ cash/debit card
☐ iPad	☐ receipt book

Part I. In-store collection procedures

- Step 1: For the purchase process, the research team faces the cigarette display and requests to purchase one of each different available cigarette package. The cigarettes are acquired starting from the top lefthand side of the cigarette display. It is the job of Person 1 to ensure that each available cigarette pack is being acquired.

- Step 2: It is essential that the price for each pack is matched and captured with the name of the pack. Each pack should be placed into an individual plastic sandwich bag with unique ID label (refer to Appendix A – Unique ID Structure Instruction Guide). The name and price are recorded on the label. If it is not possible to record the information while in the store, the audio recorder is used to capture the pack name (along with pack descriptors) and price; additionally, the packs are lined up in the carrying bag in the order they are listed on the receipt, until there is an opportunity to record the name and price on the label.
    - For retailers where the price is not given via bar code, staff asks for the price that the retailer typically sells the pack for in order to avoid receiving a "discounted" price due to purchasing a large quantity of cigarette packs.

- Step 3: Person 1 asks the merchant to confirm that one pack of each type of cigarette they sell has been acquired. After this is confirmed, the entire purchase is paid for and the receipt is retained. The receipt is often used for checking price data and it is also submitted for reimbursement. If the retailer does not provide receipts, a receipt book is used and the price for each cigarette pack is recorded before leaving the store.

- Step 4: After data collection and payment are complete, staff ask the merchant if it would be possible to take a photo of the retail display and if allowed use the Sony camera to take the picture.

- Step 5: Once back in the car, the doForms app on the iPad is opened and details about the purchase are entered including: the city, neighborhood socioeconomic group, neighborhood ID, date, type of vendor, number of packs collected and number of stores visited in neighborhood. At the end of the day of field work when there is internet access, the data are uploaded from the doForms app (refer to Appendix B - doForms Instruction Guide).

Part II. Data consolidation (initial vendor)

Objective: Create a photographic archive of packs purchased at the initial store to facilitate data collection at subsequent stores. Each pack of cigarettes is photographed and placed into a "brand family folder" on the iPad.

Procedures

After purchasing the cigarettes and placing them into individual Ziploc baggies with a label, a photograph is to be taken of the "front" of each cigarette pack. The iPad is used take a picture of each individual cigarette pack in its plastic bag.

- Step 1: Ensure that the brand name of the cigarette and its price is written on the baggie label.

- Step 2: A photo of the pack in the baggie is taken from above, parallel to the pack, without casting a shadow over the pack, with all four corners in the shot, in focus, and with the ID label clearly displayed.

- Step 3: Each photo is placed into a brand family folder/album on the iPad. The research team discuses and determines relevant brand families based on knowledge of the local cigarette market.
    - To create a new folder, press the “plus” icon in the upper righthand corner of the photos app on the iPad. Rename the folder according to the brand group name. Repeat this process for every brand.

- Step 4: The brand family folders/albums are alphabetized.

- Step 5: All of the cigarettes are returned to the appropriate labeled carrying bag until archival imaging occurs.

Subsequent Sites Collection (time in each store approx. 30 minutes)

Objective: Visit **35** vendors. Acquire one of every type of cigarette pack that was not already purchased at a previous vendor.

Equipment:	☐ Sony camera
☐ Sony audio recorder	☐ writing pens
☐ Ziploc sandwich bags	☐ carry bag
☐ ID labels	☐ cash/debit card
☐ iPad	☐ receipt book

Part I. In-store collection procedures

- Step 1: For the purchase process, the research team faces the cigarette display and starts at the top lefthand corner of the display to match each pack of cigarettes with the images from the previous store/s in the iPad folders. Any cigarettes that are not in the folder are purchased. Both Persons 1 and 2 will be involved in this procedure.

- Step 2: It is essential that the price for each new pack is matched and captured with the name of the pack. Each pack is placed into an individual plastic sandwich bag with unique ID label. The name and price are recorded on the label. If it is not possible to record the information while in the store, the audio recorder is used to capture the data by stating the pack name (along with pack descriptors) and price; additionally, the packs are lined up in the carrying bag in the order they are listed on the receipt, until there is an opportunity to record the name and price on the label.

- Step 3: Person 1 asks the merchant to confirm that one pack of each type of cigarette they sell has been acquired. After this is confirmed, the entire purchase is paid for and a receipt retained that will be submitted for reimbursement. If the retailer does not provide receipts, the receipt book is used to record the price for each cigarette pack before leaving the store.

- Step 4: After data collection and payment are complete, staff ask the merchant if it would be possible to take a photo of the retail display and if allowed use the Sony camera to take the picture.

- Step 5: Staff open the doForms app on the iPad and enter details about the purchase including: the city, neighborhood socioeconomic group, neighborhood ID, date, type of vendor, number of packs collected and number of stores visited in neighborhood. At the end of the day of field work when there is internet access, staff upload the data from the doForms app (refer to Appendix B – doForms Instruction Guide).

Part II. Data consolidation (subsequent vendors)

Objective: Update the photographic archive of packs purchased at subsequent stores to facilitate additional data collection. Each new pack of cigarettes is photographed and placed into a "brand family folder" on the iPad. This procedure is repeated for each vendor.

Procedures

Repeat the "initial vendor" data consolidation procedures

- Step 1-5: Repeat previous data consolidation procedures.

- Step 6: After data collection is completed in the final city, upload all images of the front of the packs, archived on the iPad, to Dropbox.

PRODUCT INVENTORY, ARCHIVAL IMAGING AND UPLOADING DATA

Objective: Generate a product inventory of each cigarette pack and images of each pack in a uniform manner, save and label them, and secure them on an online database.

Equipment:		
☐ Sony camera	☐ N size battery charger	☐ 2 Studio LED lights
☐ ScanDisk 16G memory card	☐ camera case	☐ computer
☐ Kingston memory reader	☐ 2 Sony N size batteries	☐ Promaster mini tripod
☐ turntable	☐ 2 light covers	☐ 16 GB drive
☐ light box	☐ 3 travel adaptors	☐ Dropbox application
☐ cigarette packs	☐ 2 light stands	☐ REDCap online form

Part I: Creating product inventory

Procedures

An online data entry form is completed for each tobacco package collected, using REDCap, an online data entry tool. Entry begins with packs purchased from store 1 and data are entered **ONE** pack at a time. For each pack complete an entry form with the following product details: unique ID number, brand name, description of pack, manufacturer name, number of sticks per pack, price, type of tobacco product, type of retailer where product was purchased, place of industrialization and which shipping box the pack was placed into.

- Steps 1-7: Presented in REDCap Product Inventory Training
  Appendix C - REDCap Product Inventory Instruction Guide

Part II: Archival imaging and uploading

Procedures

At least nine images of each tobacco product purchased are generated. These images are then saved, labeled and uploaded to the shared Dropbox account.

- All Steps: Presented in Imaging Training
  Appendix D – Imaging Instruction Guide

PACKAGING AND SHIPPING

Objective: Properly package cigarette packs for shipment and support shipping logistics.

Equipment:	
☐ cigarettes	☐ Commercial Invoice Form*
☐ standard shipping boxes	☐ JHU Letter of Intent*
☐ packaging tape	☐ Personal Effects CF 3299 Form*
☐ packaging popcorn foam	☐ product inventory form*

*These documents are required for Customs clearance and will be provided by IGTC once the packs are ready for shipment.

Part 1: Packaging and shipping

Procedures

A packaging/shipping store is visited to identify shipping material and the best size and type of box to use for shipping. Staff tests for exactly how many cigarettes can fit into the box with shipping material. If more than one box is required to ship all of the packs, label each box with a numeric value 1-10 as needed.

Once all images are completed, equipment items are shipped back to IGTC in Baltimore, USA.

- Step 1: Identify and purchase box for the equipment suitcase and place suitcase in box.

- Step 2: Line the box containing the cigarette packages with the protective wrapping material. Place cigarette packs into the box with baggie label facing forward.

- Step 3: Email the IGTC Project Manager, Carmen Washington, cwashin8@jhu.edu, when the cigarette packs and the equipment are ready for pickup, and support shipping logistics.
