# Supplementary material for: The Tobacco Pack Surveillance System: A Protocol for Assessing Health Warning Compliance, Design Features, and Appeals of Tobacco Packs Sold in Low- and Middle-Income Countries
Source: JMIR Public Health Surveill. 2015 Aug 12;1(2):e8. doi: 10.2196/publichealth.4616 (PMC4869212; doi:10.2196/publichealth.4616)
Supplement: Multimedia Appendix 2 [file publichealth_v1i2e8_app2.pdf]

JOHNS HOPKINS
BLOOMBERG SCHOOL
of PUBLIC HEALTH

Institute for Global Tobacco Control

Appendix 2: In-field Data Capture Form for Vendor and Neighborhood

About doForms

doForms is an iPad application used for mobile data collection. For the purposes of the Tobacco Pack Surveillance System (TPackSS) project, doForms is used to enter information about tobacco product purchases while in the field. doForms makes it possible to enter and save information in the field and send the stored information to the IGTC account once you have internet access.

Entering and Saving Data with doForms

- Step 1: Open the doForms app on the iPad.

- Step 2: Click on “Forms.” The form may take a few seconds to load.

- Step 3: Follow the instructions below, which correspond with each data entry field, to complete the form.
  A. *City*- Identify the relevant city from the three choices provided.
  B. *Neighborhood SES*- Identify the relevant neighborhood SES from the three choices provided (e.g. low, middle, high).
  C. *Neighborhood ID*- Identify the relevant neighborhood from the four choices provided.
  NOTE: This field will not auto-update. Therefore, it is very important to keep track of neighborhood IDs during the course of the field work so that IDs are not duplicated or omitted in doForms.
  D. *Date visited*- Enter the date, including the month, day and year (e.g. January 1, 2013).
  E. *Type of vendor* (e.g. *supermarket, bakery, kiosk*) - Enter the type of vendor from which tobacco products are purchased.
  F. *Packs collected*- Enter the number of packs purchased. If no packs are collected enter 0.
  G. *Vendors visited in each neighborhood*- Identify how many vendors are visited in each neighborhood, including the current vendor. The minimum number of stores visited should be one and the maximum should be four.

- Step 4: Once you have entered ALL information, click “Save as Complete.”

- Step 5: Repeat Steps 1-4 for every store visited.

- Step 6: Information should be sent to the doFORMS app at the end of a day of field work when there is internet access. Select all forms that appear and click “Send Selected Form(s)” at the right bottom corner of the page. Sending items may take a few moments. Once forms are successfully sent, a message will appear.

- Step 7: Close the doForms app.
