# Supplementary material for: The Tobacco Pack Surveillance System: A Protocol for Assessing Health Warning Compliance, Design Features, and Appeals of Tobacco Packs Sold in Low- and Middle-Income Countries
Source: JMIR Public Health Surveill. 2015 Aug 12;1(2):e8. doi: 10.2196/publichealth.4616 (PMC4869212; doi:10.2196/publichealth.4616)
Supplement: Multimedia Appendix 3 [file publichealth_v1i2e8_app3.pdf]

Appendix 3: REDCap In-country Inventory Form

About REDCap

REDCap is an online data collection tool, and it will be used to manage tobacco pack inventory.

Logging In

Prior to being required to create a tobacco pack inventory, the primary REDCap user (the staff member responsible for inventory) will receive an email from a REDCap administrator containing a link to the TPackSS project, a username and password. A prompt to change the password will appear upon first login. Ensure that all staff responsible for entering inventory information for your project, as well as the project director, have both the username and password. In the case that the password is forgotten, REDCap will only send an email to reset the password to the primary user's account.

- Step 1: Enter REDCap by following the link and providing the username contained in the original email and your newly created password.

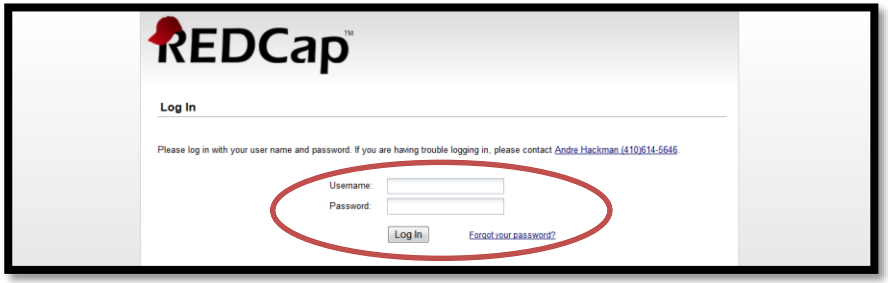

Project Page

- Step 2: Once logged in, the TPackSS project Setup page will appear. Proceed to click on the Project Home tab, located to the left of the Project Setup tab.

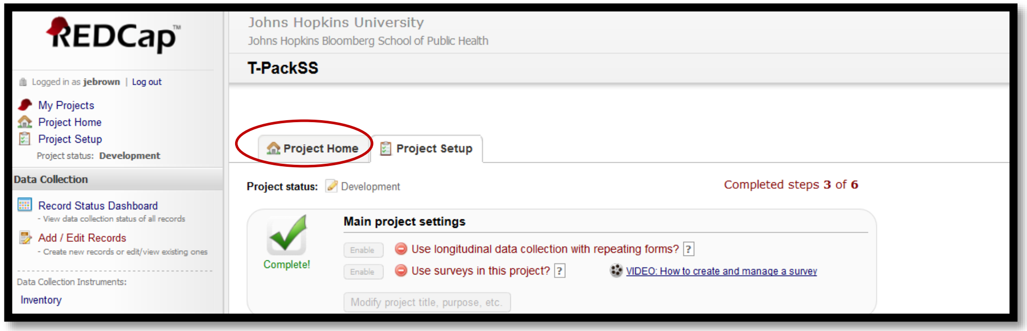

Project Home Page

On the Project Home page, the project title will appear on the top, the menu on the left and the main viewing area on the right. On the Project Home page, the list of current users and project statistics can be viewed. Project statistics will show the total number of records entered by users, as well as "In group" records. You have already been assigned to a group, based on your country. "In group" records will show the number of records that you enter as an organization. You will only be able to view and edit "In group" records.

At the top of the menu, quick links to commonly used pages can be found. The "Data Collection" section below is devoted to data entry. This is the section you will use most frequently.

- Step 3: Proceed to click on "Inventory" underneath "Data Collection" instruments to access the Inventory page.

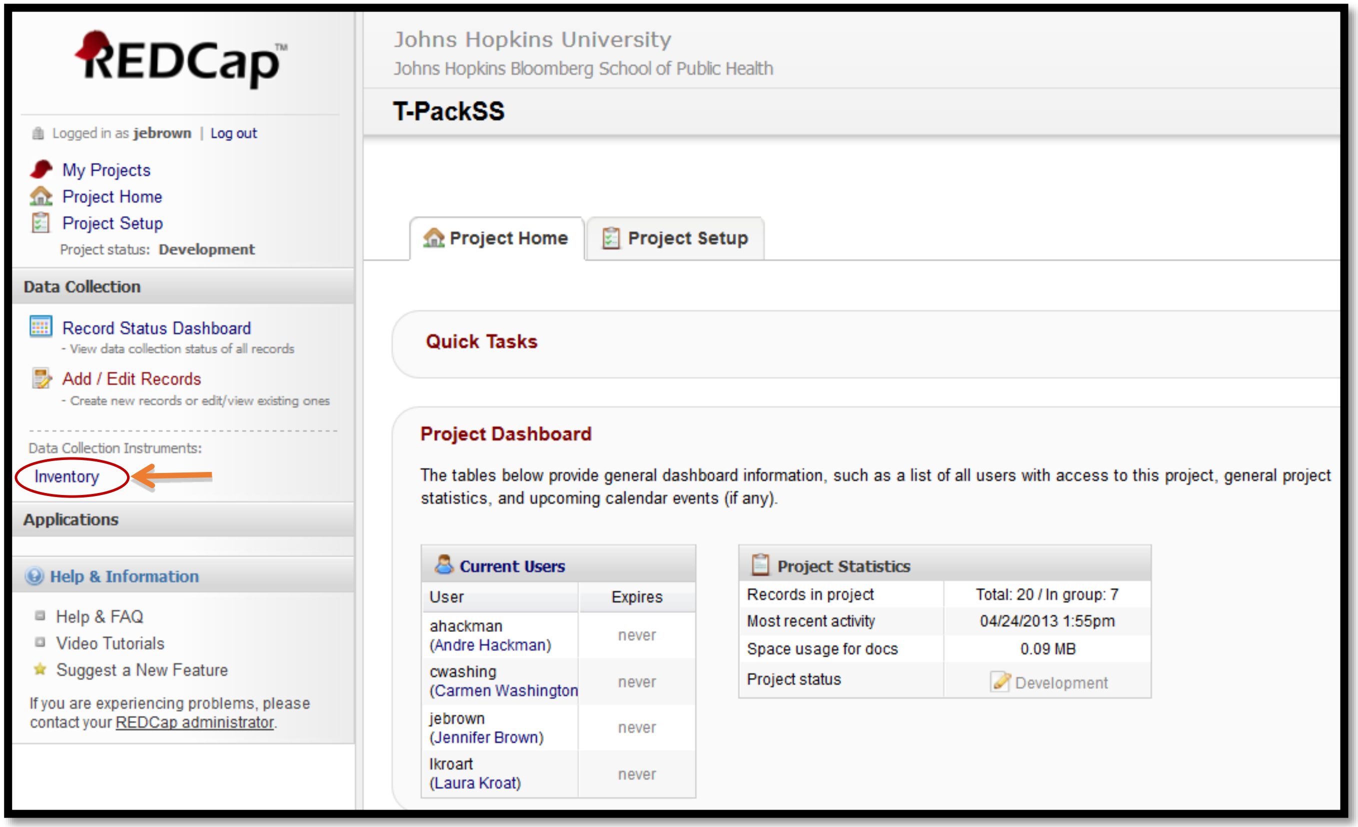

Inventory Page
On the Inventory page, the total number of records created will appear at the top.

A drop-down menu will appear for “Incomplete Records” and “Complete Records.” When a record (previously created and saved) is clicked on, the data entry form associated with that record will appear. Information on the data entry form can then be edited.

- Step 4: In order to create a new record, enter a Unique ID in the text box field where instructed to “Enter a new or existing Unique ID” and click Tab or Enter. A data entry form associated with that Unique ID will appear.

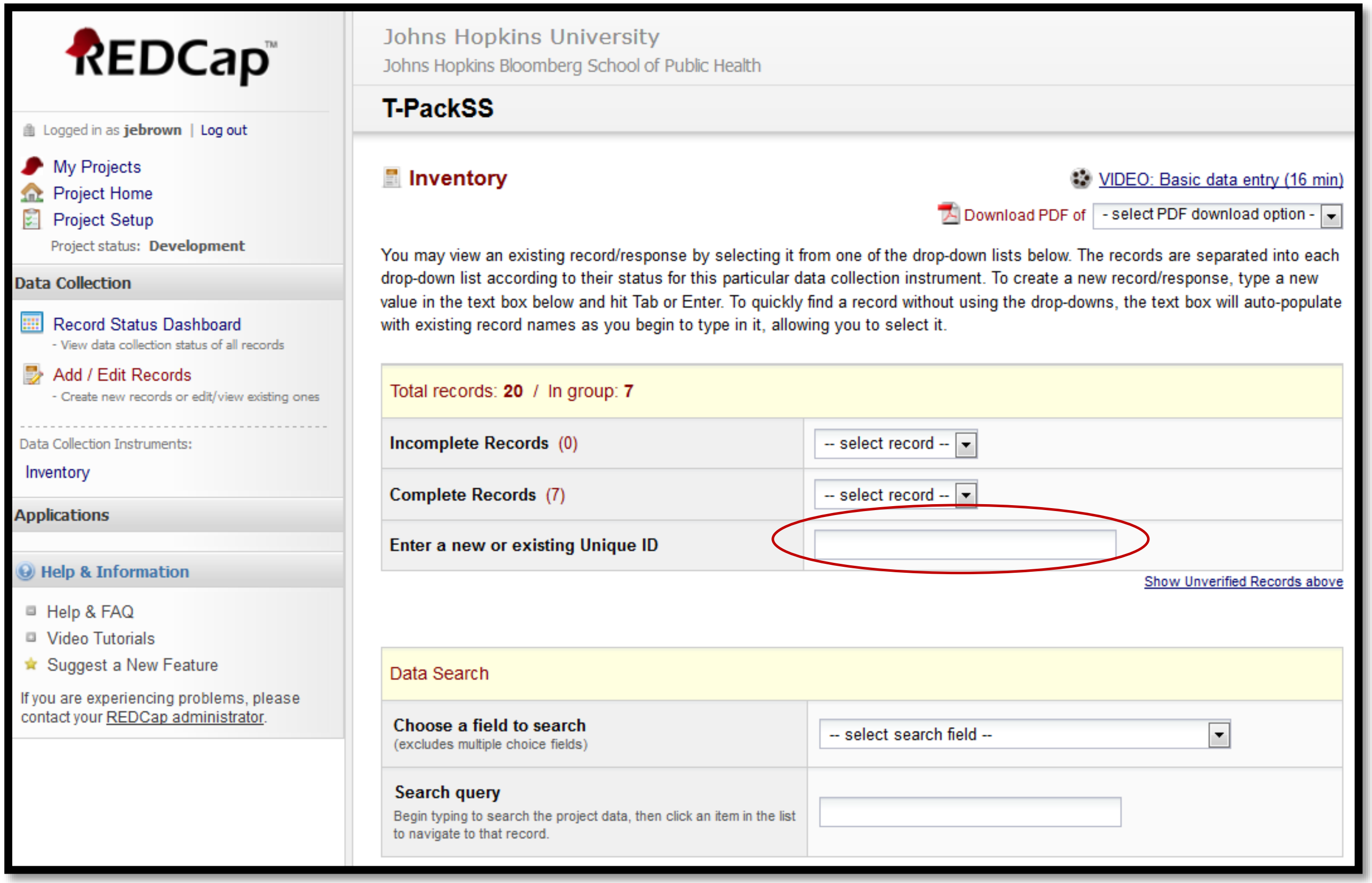

Inventory Data Entry Form

The "Inventory" data entry form is the page where you will enter all the required information associated with a particular tobacco product.

- Step 5: Follow the instructions below, which correspond with each data entry field, to complete the data entry form.
  A. *Name of Brand*- Enter name of brand as it appears on the pack, including the title and any descriptor (e.g. Marlboro Lights). If the name of brand appears in two different types of characters on the pack (e.g. Chinese characters AND Roman characters), enter the brand in non-Roman characters in this field and proceed to the next field.
  B. *Romanization of Name of Brand*- If the name of brand was entered in non-Roman characters in the "Name of Brand" field but also appears in Roman characters on the pack, enter the brand in Roman characters in this field. If the name of brand only appeared in non-Roman characters, enter the transliteration of the name of brand. If this field does not apply (e.g. name of brand only appeared in Roman characters on the pack and was previously entered in "Name of Brand" field), enter "n/a" and proceed to the next field.
  C. *Short Description of Pack (e.g. hard pack vs. soft pack, how appearance differs from similar packs)*- Enter a short description of the pack, noting whether it is a hard or soft pack. If the appearance of the pack is similar to another pack in the collection, include a description of what distinguishes it from the others (e.g. hard pack, maroon top, white package, kretek mild).
  D. *Manufacturer* (e.g. Philip Morris International)
  E. *Sticks per Pack*- Only numbers may be entered in this field (e.g. 20).
  F. *Price (in local currency)*- Only numbers may be entered in this field (e.g. 1.23).
  G. *Type of Product*- Must choose from pre-designated classifications in drop-down menu.
  H. *Type of Store (e.g. supermarket, kiosk, gas station, bakery)*
  I. *Place of Industrialization (country pack was manufactured in)*
  J. *Box Number (box that pack is being shipped in)* - Only numbers may be entered in this field (example: 1).
  NOTE: If any information requested on the data entry form cannot be found on the pack, enter "not indicated". Do not guess and do not leave any fields on the data entry form blank.

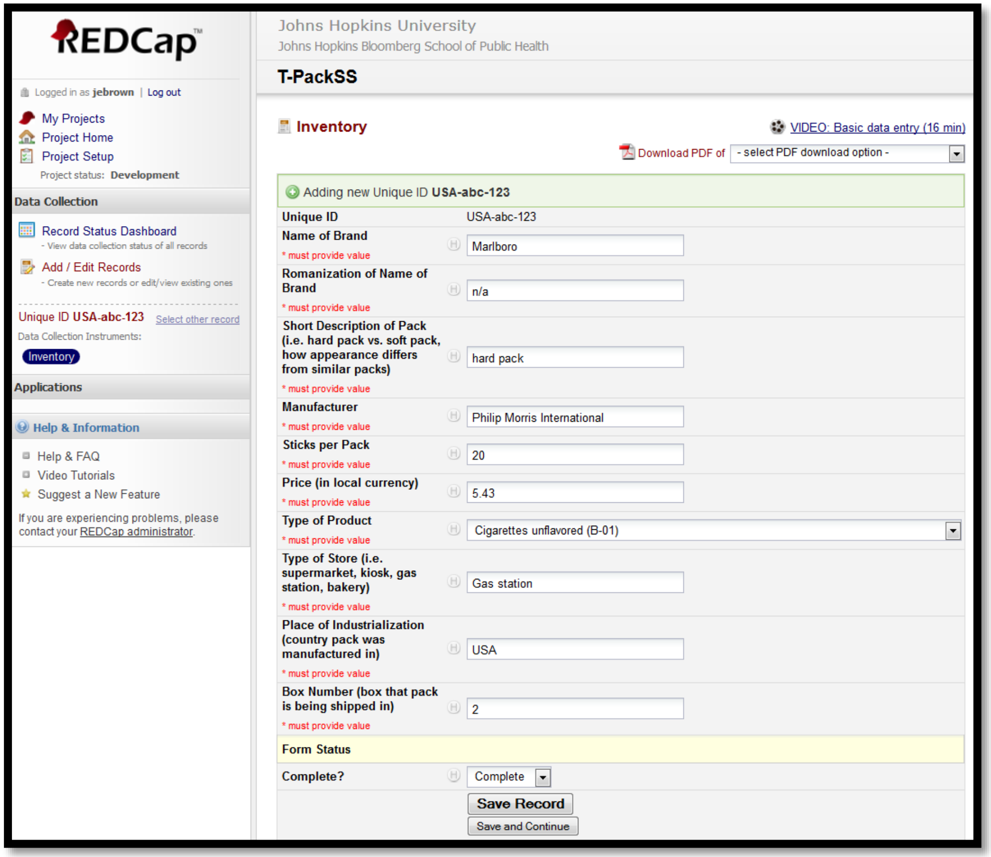

- Step 6: Once all information is entered on the data entry form, click "Complete" from the drop-down menu at the bottom of the form.
- Step 7: Click on the "Save record" button. The inventory home page will automatically appear.
- Step 8: Repeat Steps 1-7 until one data entry form has been completed for every tobacco product collected.
- Step 9: Notify your IGTC staff coordinator, Carmen Washington (cwashin8@jhu.edu) or Jennifer Brown (jbrow212@jhu.edu), when data entry is complete. IGTC will then be able to generate an inventory list.
