# Supplementary material for: The Tobacco Pack Surveillance System: A Protocol for Assessing Health Warning Compliance, Design Features, and Appeals of Tobacco Packs Sold in Low- and Middle-Income Countries
Source: JMIR Public Health Surveill. 2015 Aug 12;1(2):e8. doi: 10.2196/publichealth.4616 (PMC4869212; doi:10.2196/publichealth.4616)
Supplement: Multimedia Appendix 4 [file publichealth_v1i2e8_app4.pdf]

## Part 1: Producing Images

Our overall goal is to generate images of the packs that are the highest possible quality by following uniform protocol. The most important part of this step is to generate images that are clear, well-lit and have all four corners of the pack in the photo.

---

*Setup and Assembly:*

See photos on page 2 for setup reference.

- Step 1: Set up the light tent.
  - A. Unzip the light tent and carefully unfold.
  - B. Attach the white backdrop using the Velcro so that it covers both the back panel and the bottom of the light tent.
  - C. Place the turntable in the middle of the box, turned so that the "0" mark is in the front and centered, lined up with the red dot.

- Step 2: Place camera on table tripod and screw into place.
  - A. Bend out the legs of the tripod so that they are fully extended.
  - B. Ensure that the lens is focused at the center of the turntable.
  - C. Make sure the camera is set to "AUTO" mode.

- Step 3: Set up the lighting.
  - A. Remove the lights, stands and light covers from the suitcase.
  - B. To connect the light to the light cover, match up the three brass tabs on the light with the three slots on the light cover, and rotate them into place. Tighten the knob on the top of the light cover to secure it.
  - C. To connect the light to the stand, set the hole on the base of the light onto the silver knob on the stand.
  - D. Plug in the lighting, and turn the dial switch on the back of each light as far as they will turn.
  - E. Place the lights at the two front corners of the light tent, as shown. Lights should be placed on a diagonal.

---

*Setup Overview:*

The lights should be placed at the front corners of the light tent, as shown.

Angle the lights so that the light projects through the sides of the tent.

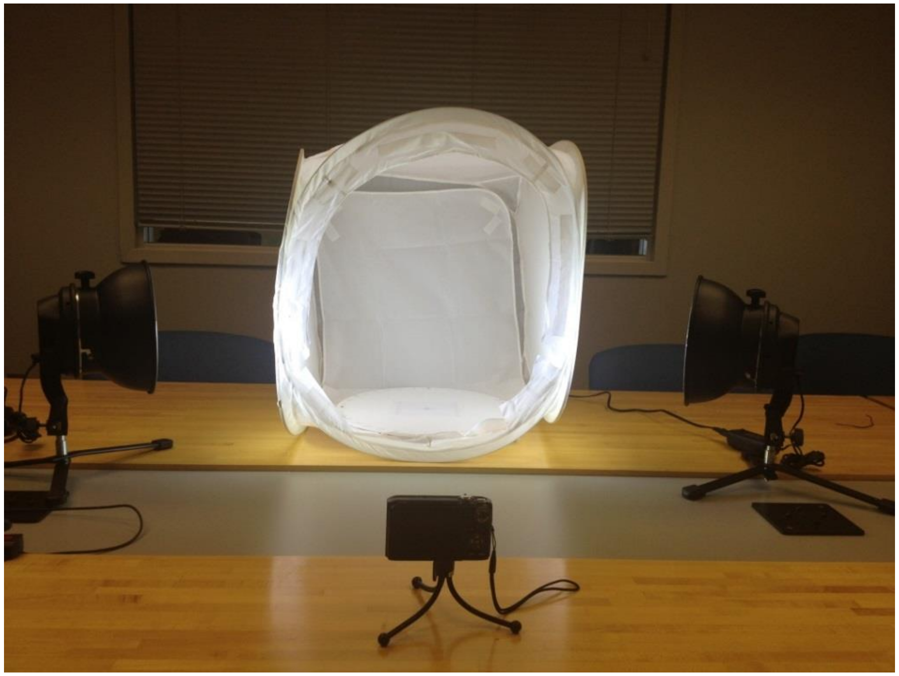

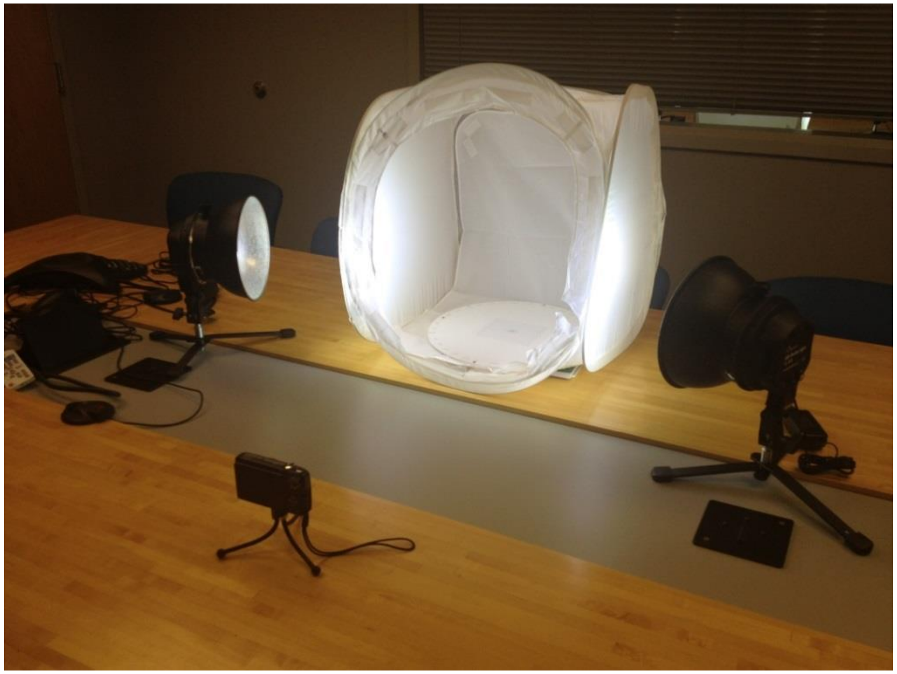

*Photographing Packs:*

**There are four standard for high-quality images that, if met, will prevent reshoots:**

- **Packs are photographed straight-on.**
- **All four corners of the pack are in the shot.**
- **The photo is in focus and well-lit.**
- **Zoom: If necessary, use the zoom feature to get closer to the pack, but make sure the image does not go out of focus.**

Use the chart on page three as a guide for photographing each pack, keeping the following in mind:

A. In order to keep track of the packages, photograph ten packs at a time, upload them, then photograph 10 more.
    - Be sure to replace the pack back into its correct plastic bag after photographing.

B. **Important!**
    If there is anything printed on the **cellophane** itself,
    - Take two pictures of the pack in the cellophane with the printed image/text in view —one picture of the front of the pack at 20 degrees and one picture of the front of the pack on a 0 degree angle. If the design differs on any other side of the pack, photograph these sides as well.

    If there is a larger package on the primary package,
    - Take a picture of the larger package in a series of five photos in the following order: 0, 90, 180, 270 and 20 degrees.

    Afterwards,
    - Carefully remove the cellophane and/or larger package and begin with the nine shots outlined in the following chart. Place the cellophane in the labeled bag. It is important to remove the cellophane because it creates a glare when photographed.

C. To begin, pack should be placed with the front facing the camera, with the edge of the front of the pack on the black line.

D. Photos **must be taken in the order on the following chart** so that IGTC staff will be able to discern between different sides.

E. Keep the chart on page 4 open while photographing packs for reference.

**Standards for high-quality images:**

- **Packs are photographed straight on.**
- **All four corners of the pack are in the shot.**
- **The photo is in focus and well lit.**
- **If necessary, use the zoom feature, but make sure the image is in focus.**

**Photos MUST be taken in the following order:**

| # | Side | Instructions | Example |
|---|---|---|---|
| 1<br>2<br>3<br>4<br>5 | Front on 20 degree angle<br>Front (0 degrees)<br>Right side (90 degrees)<br>Back (180 degrees)<br>Left side (270 degrees) | Check the pack for printing on the cellophane. If there is printing, take two photos with the cellophane: the front of pack at 20 degrees, then at 0 degrees. If there is another type of cover on the pack, photograph in the following order with the specified angle: 20, 0, 90, 180, and 270 degrees.<br><br>Remove the cellophane and rotate the turntable so that the "20" lines up with the red dot.<br><br>Photograph all four sides, in the following order: 1) front on 20 degree angle, 2) front at 0 degree angle, 3) right side (90 degrees), 4) back (180 degrees), and 5) left side (270 degrees). | 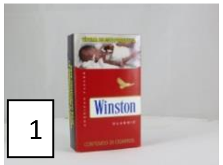  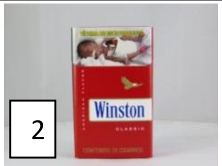  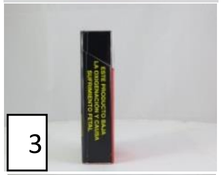  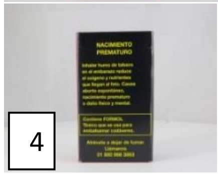  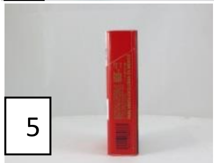  |
| 6 | Top | Zero the turntable. Place the pack face down, lined up with the black line. If there is text on the top of bottom, make sure it is right-side up and readable. | 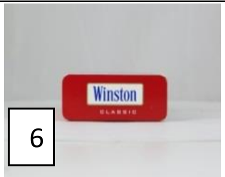  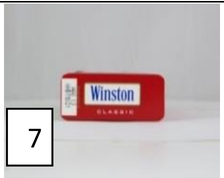  |
| 7 | Bottom | | |
| 8 | Front, open, on 20 degree angle | Open the pack and place it on the black line, front facing forward. Rotate the turntable so that the 20 lines up with the red dot. | 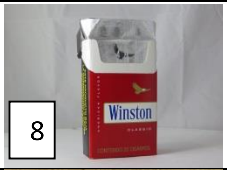  |
| 9 | One cigarette | Place the provided cardboard inside the light tent. Remove one cigarette and place it on the cardboard. Pick up the camera and tripod and photograph the cigarette from a few inches above. | 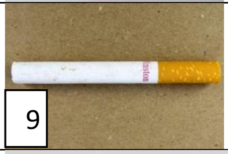  |
| 10 | Other | *If there are any other aspects of the pack such as writing on the inside of the flip-top, photograph these last. | 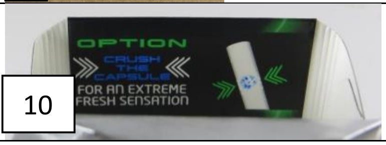  |

**Part Two: Uploading to Dropbox**

- Step 1: Remove the memory card from the camera, insert the memory card into the reader and then into a USB port.

- Step 2: Open the Dropbox folder on your computer (Start›My Computer›Dropbox) or on www.dropbox.com.
  A. Navigate: appropriate country folder›"Tobacco Pack Images"›appropriate city folder.
  B. IGTC Staff has already created folders for each neighborhood. Open the folder for the corresponding neighborhood (ex: H1).

- Step 3: Create a new folder that corresponds with each unique ID number as you upload the corresponding pack images.
  A. Create a new folder for the first set of images, using the pack's unique ID. (ex: THA_BAN_H1_001)

- Step 4: Open a new Finder window, and open the files from the memory card.
  A. View as thumbnails.
  B. Copy and paste the corresponding series of images into the labeled folder.
  C. Rename the files in Dropbox by highlighting the series of pictures and renaming the first image to match the folder name (ex: THA_BAN_H1_001). All subsequent images should automatically be renamed (ex: THA_BAN_H1_001 (1), and so forth).
  **Note**: If you are using dropbox.com, rename images following the same instructions but do so BEFORE uploading the series of images to the corresponding folders on dropbox.com.
  D. Repeat this process for every new pack.

- Step 5: Feedback.
  A. After the first 10 packs have been photographed and uploaded to Dropbox, contact the IGTC team and notify the appropriate staff person that photographs have been uploaded and the location where they are saved. IGTC staff will review the photographs uploaded to Dropbox and provide feedback.
  B. After IGTC staff has provided feedback, reshoot and rename the existing photographs as necessary and proceed with taking new photographs.
  C. On every day that packs are photographed and uploaded to Dropbox during post-field work, email IGTC staff and provide a summary of work completed. In the email, include the photographs taken and which folders they were uploaded to (ex: Today, THA-BAN-H1, folders 001-049 were uploaded.), the total number of packs that were collected in the city whose packs are being photographed, and the total number of packs that have been photographed for that particular city (ex: 49 packs of a total of 156 packs collected in Bangkok have been photographed to date.).

***Important:** Dropbox will only upload the photos while the computer is on and connected to the internet. Allow time for the photos to sync with Dropbox before shutting down the computer and logging out.
