# Supplementary material for: The Tobacco Pack Surveillance System: A Protocol for Assessing Health Warning Compliance, Design Features, and Appeals of Tobacco Packs Sold in Low- and Middle-Income Countries
Source: JMIR Public Health Surveill. 2015 Aug 12;1(2):e8. doi: 10.2196/publichealth.4616 (PMC4869212; doi:10.2196/publichealth.4616)
Supplement: Multimedia Appendix 5 [file publichealth_v1i2e8_app5.pdf]

Turkey Codebook for 2013 Data Collection

Please refer to the Excel document "TPackSS HWC_Codebook questions and regulations" for the laws from which specific questions are derived. If you feel that some aspect of the pack warrants further discussion, make a note in the "topics for further discussion" section.

Unique ID (unique_id)

Enter the Unique ID that is printed on the label on the bag in which the pack is held.

1. Type of Product (product_type)

What type of product is this?

[Definition of "tobacco products" (Law No. 25692, Article 4): "All products that are smoked, sniffed, sucked or chewed that are manufactured from normal or genetically modified tobacco leaf."]

Refer to the front of the pack to answer this question. If the type of product is not written on the front of the pack, refer to the back and if not found on the back, the sides of the pack. If there are two descriptions of the product found on the pack, choose the more specific classification (e.g. when "clove cigarettes" AND "Class A cigarettes" are written on a pack, the product will be classified as "clove cigarettes"). If product type is not written on the package, look at the sticks inside the package and refer to additional information online. In your online search for information, refer only to credible sources (official brand websites, government registries of tobacco products or Euromonitor).

[Definition of "front side of pack" (Law No. 4721, Article 3): "The term 'the most visible, broad front side' means the opening side of a hard pack or the most visible side of a soft pack." For the purposes of defining the front side of the pack for soft packs, refer to the location of the pictorial health warning which should be located on the front of the pack.]

(1) Manufactured cigarettes
(2) Bidis
(3) Cloves or Kreteks
(4) Cigarillo

☐ Check here if you used additional online information to determine product type. (product_type_info)

Enter your source here. (product_source)

____

Section A: Front of Pack

A1. Presence of warning (front_warn_presence)

Is there a health warning label on the front of the pack?

(1) Yes
(0) No

If "No", skip to Section B.

A2. Warning content (front_warn_content)

Using the Appendix, indicate the number of the health warning that appears on the package. If there is no warning that matches the warning on the pack select "No match".

	1 (1)	2 (2)	3 (3)	4 (4)	5 (5)	6 (6)	7 (7)	8 (8)	9 (9)	10 (10)	11 (11)	12 (12)	13 (13)	14 (14)	No Match (99)
Warning content															

A3. Location of HWL (front_warn_loc)

Does the health warning start from the end of the lid or the finishing line of the tax stamp?

(1) Yes
(0) No

A4. Parallel HWL (front_warn_parallel)

Is the health warning parallel to the top edge of the surface it is located on?

(1) Yes
(0) No

A5. Direction of HWL (front_warn_dir)

Is the health warning information printed in the same direction as the other information printed on that side of the pack?

(1) Yes
(0) No

A6. HWL is indelible (front_warn_indelible)

Is the health warning printed in a manner that it cannot be removed?

(1) Yes
(0) No

A7. HWL visibility (front_warn_visible)

Is the health warning visible with the lid open?

(1) Yes
(0) No

A8. HWL unobstructed (front_warn_unobstruct)

Is the health warning placed so that other labels, tax stamps, price tags or any other components do not intervene with the textual and/or visual components of the warning?

(1) Yes
(0) No

A9. HWL integrity (front_warn_integrity)

Does the warning appear as it does in the Appendix in regards to format, proportions, colors and graphical integrity?

[When required by the size of the package, the warning, corresponding text, photograph, graphics or pictures may be moved to the right side or to the bottom area. Law No. 4721 permits the TAPDK Committee to make these determinations when evaluating applications.]

(1) Yes
(0) No

A10. HWL language (front_warn_lang)

Is the health warning text written in Turkish?

(1) Yes
(0) No

A11. HWL letter case (front_warn_ltrcase)

Is the health warning text in lowercase letters, except for the first letter which should be capitalized?

(1) Yes
(0) No

A12. HWL spacing (front_warn_txtspc)

Is the gap between the rows of health warning text equal to the size of the font?

The gap between the rows can be eyeballed except in cases where you think the pack may be noncompliant. In these cases, measure the font size and gap size using a font ruler.

(1) Yes
(0) No

A13. HWL left justified (front_warn_leftjust)

Is the health warning text left justified?

(1) Yes
(0) No

A14. HWL word breaks (front_warn_wordbreak)

Are the words within the health warning text not separated by a hyphen sign or split across lines?

(1) Yes
(0) No

A15. HWL font (front_warn_font)

Is the health warning text written in Helvetica font?

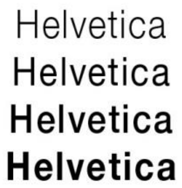

Image 1. Sample of Helvetica font

(1) Yes
(0) No

A16. HWL frame (front_warn_frm)

Is the health warning framed with a black line?

(1) Yes
(0) No

If 'No', skip to A18.

A17. HWL frame size (front_warn_frmsize)

Is the health warning frame 3-4mm in width?

(1) Yes
(0) No

Measurement

- Complete the following pack measurements IN CENTIMETERS (cm) and round all measurements to the nearest tenths place (e.g. .12 becomes .1). Enter the measurement to the first decimal place.
- When measuring a package with beveled edges measure the face of the package only, not including the beveled portion.
- When measuring a package with rounded edges measure the face of the package only, not including any portion of the pack that is not flat.
- When measuring a soft pack measure only the face of the tobacco package and do not include ANY of the foil area.
- Measure the text in an upright position, following the direction of the text.
- When measuring the width, measure across the points at the bottom edge of the pack.
- Include the border in your measurement of the warning.

A18. HWL height (front_warn_height)

Enter the height (cm) of the health warning label. ______

A19. HWL width (front_warn_width)

Enter the width (cm) of the health warning label. ______

A20. Height (front_height)

Enter the height (cm) of the front panel of the pack. ______

A21. Width (front_width)

Enter the width (cm) of the front panel of the pack. ______

A22. Percentage of front of pack covered by warning (front_warn_percent)

Using the measurements above, the percentage of area of the front panel of the pack covered by the health warning label will be calculated automatically. _____

A23. Edges of pack (front_bvlorrnd)

Does the front of the pack have beveled or rounded edges?

Packs are only considered to have beveled or rounded edges when they are hard packs. Refer to Images 2 and 3 for examples. Choose N/A if a pack is a soft pack, cylindrical pack or a tin pack.

Example:

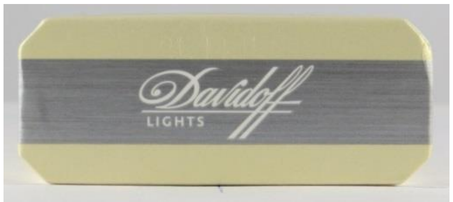

Image 2. Beveled edges

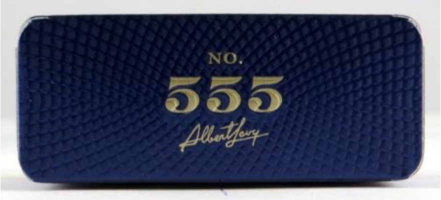

Image 3. Round edges

(1) Yes
(0) No
(9) N/A

If ‘No’ or ‘N/A’, skip to Section B.

A24. Health warning label coverage (front_bvlorrnd_coverage)

Does the health warning label cover the beveled or rounded edges of the pack?

(1) Yes
(0) No

<u>Section B: Back of the Pack</u>

B1. Presence of warning (back_warn_presence)

Is there a health warning label on the back of the pack?

(1) Yes
(0) No

If “No”, skip to Section C.

B2. Warning content (back_warn_content)

Using the Appendix, indicate the number of the health warning that appears on the package. If there is no warning that matches the warning on the pack select "No match".

	1<br>(1)	2<br>(2)	No Match<br>(99)
Warning content			

B3. Parallel HWL (back_warn_parallel)

Is the health warning parallel to the top edge of the surface it is located on?

(1) Yes
(0) No

B4. HWL is indelible (back_warn_indelible)

Is the health warning printed in a manner that it cannot be removed?

(1) Yes
(0) No

B5. HWL visibility (back_warn_visible)

Is the health warning visible with the lid open?

(1) Yes
(0) No

B6. HWL unobstructed (back_warn_unobstruct)

Is the health warning placed so that other labels, tax stamps, price tags or any other components do not intervene with the textual and/or visual components of the warning?

(1) Yes
(0) No

B7. HWL language (back_warn_lang)

Is the health warning text written in Turkish?

(1) Yes
(0) No

B8. HWL letter case (back_warn_ltrcase)

Is the health warning text in lowercase letters, except for the first letter, which should be capitalized?

(1) Yes
(0) No

B9. HWL centered (back_warn_centered)

Is the health warning text centered on the back?

(1) Yes
(0) No

B10. HWL font (back_warn_font)

Is the health warning text written in black bold Helvetica font?

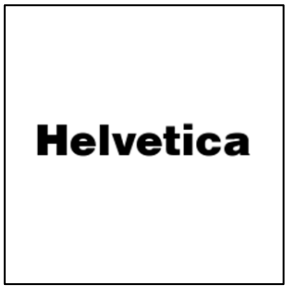

Image 4. Sample of black bold Helvetica font

(1) Yes
(0) No

B11. HWL frame (back_warn_frm)

Is the health warning framed with a black line?

(1) Yes
(0) No

If ‘No’, skip to B13.

B12. HWL frame size (back_warn_frmsize)

Is the health warning frame 3-4 mm in width?

(1) Yes
(0) No

Refer to Section A for ‘Measurement’ instructions.

B13. HWL height (back_warn_height)

Enter the height (cm) of the health warning label. ______

B14. HWL width (back_warn_width)

Enter the width (cm) of the health warning label. ______

B15. Height (back_height)

Enter the height (cm) of the back panel of the pack. ______

B16. Width (back_width)

Enter the width (cm) of the back panel of the pack. ______

B17. Percentage of front of pack covered by warning (back_warn_percent)

Using the measurements above, the percentage of area of the back panel of the pack covered by the health warning label will be calculated automatically. _____

B18. Edges of pack (back_bvlorrnd)

Does the back of the pack have beveled or rounded edges?

Packs are only considered to have beveled or rounded edges when they are hard packs. Refer to Images 2 and 3 on page 7 for examples. Choose N/A if a pack is a soft pack, cylindrical pack or a tin pack.

(1) Yes
(0) No
(9) N/A

If ‘No’ or ‘N/A’, skip to Section C.

B19. Health warning label coverage (back_bvlorrnd_coverage)

Does the health warning label cover the beveled or rounded edges of the pack?

(1) Yes
(0) No

<u>Section C: Side of the Pack</u>

C1. Presence of emission levels (side_emis_presence)

Are the tar, nicotine and carbon monoxide emission levels written on one of the sides of the pack?

(1) Yes
(0) No

If “No”, skip to Section D.

C2. Parallel emission levels (side_emis_parallel)

Are the emission levels parallel to the top edge of the surface they are located on?

(1) Yes
(0) No

C3. Emission levels indelible (side_emis_indelible)

Are the emission levels printed in a manner that they cannot be removed?

(1) Yes
(0) No

C4. Emission levels visibility (side_emis_visible)

Are the emission levels visible with the lid open?

(1) Yes
(0) No

C5. Emission levels unobstructed (side_emis_unobstruct)

Are the emission levels placed so that other labels, tax stamps, price tags or any other components do not intervene with the textual and/or visual components of the warning?

(1) Yes
(0) No

C6. Emission levels language (side_emis_lang)

Are the emission levels written in Turkish?

(1) Yes
(0) No

C7. Emission levels letter case (side_emis_ltrcase)

Are the emission levels printed in lowercase letters, except for the first letters of each content level which should be capitalized?

(1) Yes
(0) No

C8. Emission levels centered (side_emis_centered)

Are the emission levels centered on the side?

(1) Yes
(0) No

C9. Emission levels font (side_emis_font)

Are the emission levels written in black bold Helvetica font?

See Image 4 on page 9 for an example of black bold Helvetica font.

(1) Yes
(0) No

C10. Emission levels frame (side_emis_frm)

Are the emission levels framed with a black line?

(1) Yes
(0) No

If ‘No’, skip to C12.

C11. Emission levels size (side_emis_frmsize)

Is the emission levels frame 3-4 mm in width?

(1) Yes
(0) No

Refer to the ‘Measurement’ instructions in Section A.

C12. Emission levels height (side_emis_height)

Enter the height (cm) of the emission levels. ______

C13. HWL width (side_emis_width)

Enter the width (cm) of the emission levels. ______

C14. Height (side_height)

Enter the height (cm) of the side panel of the pack. ______

C15. Width (side_width)

Enter the width (cm) of the side panel of the pack. ______

C16. Percentage of front of pack covered by warning (side_emis_percent)

Using the measurements above, the percentage of area of the side panel of the pack covered by the emission levels will be calculated automatically. _____

Section D: Other

D1. Lot number (lot_number)

Is there a lot number present on the pack?

(1) Yes
(0) No

D2. Manufacturer (manufacturer)

Is the manufacturer present on the pack?

(1) Yes
(0) No

D3. Brand (brand)

Is the brand present on the pack?

(1) Yes
(0) No

D4. Product varieties (product_varieties)

Are features that distinguish product varieties manufactured under the same brand name from each other present on the pack?

(1) Yes
(0) No

D5. Quantity (quantity)

Are number of sticks printed on the pack?

(1) Yes
(0) No

D6. No sale to minors (sale_minors)

Does the phrase, "May not be sold to persons younger than 18 years old" appear on the pack?

(1) Yes
(0) No

D7. Quitline (quitline)

Does the phrase, "Hotline 171: Tobacco Quitting Consultation Line" appear on the pack?

(1) Yes
(0) No

D8. Misleading descriptors (misleading_descriptors)

Do the following misleading descriptors appear on the pack? Check all that apply.

**Misleading descriptor**	**Check here**
"Low tar" (low_tar)	
"Light" (light)	
"Ultra-light" (ultra_light)	
"Mild" (mild)	
"Extra" (extra)	
"Ultra" (ultra)	
Other (other)	

Describe "other" (misleading_other)

____
____

Section E: Topics for Further Discussion and Notes

E1. Further discussion (further_discussion)

Is there any aspect of this pack that needs further discussion?

(1) Yes
(0) No

E2. Notes on further discussion (further_discussion1)

E3. General notes (general_notes)

Note any items of interest about the pack.

