# Supplementary material for: The Tobacco Pack Surveillance System: A Protocol for Assessing Health Warning Compliance, Design Features, and Appeals of Tobacco Packs Sold in Low- and Middle-Income Countries
Source: JMIR Public Health Surveill. 2015 Aug 12;1(2):e8. doi: 10.2196/publichealth.4616 (PMC4869212; doi:10.2196/publichealth.4616)
Supplement: Multimedia Appendix 6 [file publichealth_v1i2e8_app6.pdf]

Features and Appeals Codebook for 2013 Data Collection

To begin a new record, enter the Unique ID of the pack (unique_id).

When entering text, enter the exact spelling, casing, accents and punctuation as reflected on the pack.

Section A: General Pack Information

A1. Type of product (product_type)

What is the product type?

If other product types are relevant (such as country-specific traditional tobacco products), they are to be included as options as needed.

(1) Manufactured cigarettes
(2) Cloves or Kreteks (must state "clove" or "kretek" on the pack, unless brand produces cloves/kreteks)
(3) Cigarillos (must state "cigarillo" or "cigar" on the pack, unless brand produces cigarillos)
(4) Cigarros de Palha (Straw Cigarettes from Brazil)

If additional research was necessary to clarify the product type, describe and include in text box any applicable website addresses (URL) if the internet was used for clarification. If no outside research was needed, skip this field. **(product_type_research)**

A2. Locations on side of pack (locations_listed)

Where was the pack made?

Location must be accompanied by "made in," "manufactured in," "produced in" or synonym, but can also include locations associated with a factory, as long as it is clear that it is where the product was made. This only includes locations listed on the side of the pack with manufacturer information. If both city and country are listed, list the country.

(1) Bangladesh
(2) Brazil
(3) Burma
(4) China
(5) Egypt
(6) Gulf Coast Countries
(7) India
(8) Indonesia
(9) Italy
(10) Japan
(11) Kazakhstan
(12) Korea
(13) Malaysia
(14) Mexico
(15) Nepal
(16) Pakistan
(17) Philippines
(18) Russian Federation
(19) Thailand
(20) Turkey
(21) Ukraine
(22) UAE (United Arab Emirates)
(23) United Kingdom
(24) USA
(25) Uzbekistan
(26) Viet Nam
(88) Other Country **(other_country)**
(50) London
(89) Any other city **(other_city)**
(0) No "made in" location

A3. Primary package vs. larger package (primary_vs_larger)

Is the primary pack of sticks contained in any kind of larger package?

The primary package of sticks is defined as the package in which the sticks are contained, can be closed and can stand on its own without the larger package. The larger package is defined as the package in which the primary package is contained and can be completely separated from the primary package. Cellophane is not considered a larger package.

(1) Yes, the primary package of sticks is contained in a larger package
(0) No, the primary package is not contained in a larger package **Skip to C1**

<u>Section B: Larger Package (skip this section if no larger package)</u>

B1. English on front of the larger package (english_lar)

Is there any English on the front of the pack, excluding health warnings and brand family name or brand image/crest?

For hard packs, the front of pack is the side where the flip top opens. For soft packs, the front of the pack is the side that is face up when the pack is placed so that the tobacco company text (not the health warning text) on the side of the package is upright and readable.

(1) Yes, all of the writing is in English
(2) Yes, some of the writing is in English
(0) No, none of the writing is in English
(99) Not applicable-no other writing except brand family name

B2. Pack type of the larger package (pack_type_lar)

What is the pack type of the larger package?

**Pack Type**	**Description**	**Example**
(1) Slider	Cardboard or other material that can be completely removed from the primary package.	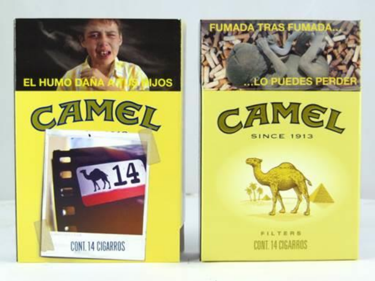
(2) Slider with fold-out	Slider style pack with a fold-out from the front or back that provides more surface area for branding.	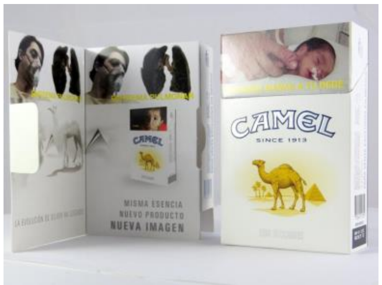
(3) Box	Pack made of tin, other metal or hard plastic, or cardboard box that contains the primary pack of sticks.	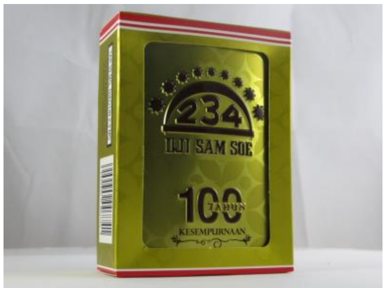
(88) Other	**(pack_type_lar_other)**	

B3. Outside fancy features of the larger pack (fancy_finish_lar)

Does the outside of the pack have any of the following features? Select all that apply:

Embossing or debossing can be tested by feeling the features of the outside of the pack.

Holographic printing is any multilayered image that changes content when viewed from different angles.

Iridescent printing refers to any image that changes color when viewed from different angles.

(1) Any embossing or debossing
(2) Shiny or metallic finish that covers the entire front (see front definition)
(3) Shiny metallic detailing
(4) Holographic text, logo or image
(5) Iridescent text, logo or image
(88) Other **(fancy_finish_lar_other)**
(0) No fancy features

B4. Change stick flavor (crush_lar)

Does the larger pack indicate in any way that the user is able to change the stick flavor (e.g. convertibles, click and roll, activate freshness)?

(1) Yes
(0) No

B5. Web Presence (web_lar)

Is there any reference to a web presence on the larger package? Select all that apply. This includes both inside and outside of the larger package.

(1) Facebook
(2) Twitter
(3) Company website
(4) Company email
(5) QR code
(88) Other **(web_lar_other)**
(0) No reference to a web presence

B6. Company phone number (phone_lar)

Is a phone number for the company listed anywhere on the larger package? This excludes phone numbers mandated by or associated with health warnings.

(1) Yes
(0) No

B7. Presence of inserts or onserts on larger package (insert_onsert_lar)

Does the larger package contain any inserts or onserts (not including those with only health warning information)? Select all that apply.

An "insert" is any communication inside an individual package and/or carton, such as a miniature leaflet or brochure. An "onsert" means any communication affixed to the outside of an individual package, such as a miniature brochure beneath the outer cellophane wrapping or glued to the outside of the stick package.

(1) Yes, the package contains an insert
(2) Yes, the package contains an onsert
(0) No inserts or onserts

B8. Indication of Stick Size (stick_size_lar)

Describe the size or sizes listed on the larger package. Select all that apply.

For other sizes, enter the English translation. Other stick sizes may include: ultraslims, the stick size written in millimeters, superlarge, etc.

(1) XL, Extra-large
(2) Kings, King or King Size
(3) Slim/Slims
(4) Superslims
(5) 100s
(6) 120s
(7) Mini, Compact
(88) Other **(stick_size_lar_other)**
(0) No size listed

B9. Colors of Larger Package (colors_lar)

Choose two of the following, looking at the entire pack.

First, select the background color of the primary package, i.e. the dominant color upon which other items are printed.

Next, select a second color. Excluding the background color and the color of the brand name text, what is the one other main prominent color (if any)? If there is no other main color, select "no other main color." The second color should be the other prominent and obvious color at first sight of the pack.

(1) Red
(2) Orange
(3) Yellow
(4) Green
(5) Blue
(6) Violet or Purple
(7) Pink
(8) Brown
(9) Black
(10) White
(11) Grey
(12) Gold
(13) Silver
(0) No other main color

B10. Indication and Color of Brand Family Name – Larger Package

The following questions are designed to capture how the brand family is indicated on the pack and the alphabet and colors in which it is written. The brand family on the pack can be denoted in two different ways: by text/number or by symbol.

A brand family name relates different varieties of products under one umbrella term, number or symbol. Examples: Marlboro, Players, 555. If the brand family is indicated by both text and/or number and symbol and/or image, select text and/or number.

Color questions prompt the list of color options below. If the brand family name is written in two or more colors, choose the color that uses the most space in the name. Iridescent text should be coded for the color of its overall sheen, usually silver. If there are multiple instances of the brand family name, choose the most prominent color.

How is the brand family name indicated on the larger package? **(brandfam_indicate_lar)**

(1) By symbol or image only.
(2) By text and/or number.

A. What is the color of the brand family symbol on the front of the pack? **(color_symbol_lar)**

(1) Red
(2) Orange
(3) Yellow
(4) Green
(5) Blue
(6) Violet or Purple
(7) Pink
(8) Brown
(9) Black
(10) White
(11) Grey
(12) Gold
(13) Silver
(99) Brand family name is indicated by text

B. What is the color of the brand family name in Roman characters or Arabic numerals? **(color_roman_text_lar)**

(1) Red
(2) Orange
(3) Yellow
(4) Green
(5) Blue
(6) Violet or Purple
(7) Pink
(8) Brown
(9) Black
(10) White
(11) Grey
(12) Gold
(13) Silver
(99) The brand family name is **not** written in Roman characters or Arabic numerals

C. What is the color of the brand family name in non-Roman characters? **(color_nonroman_text_lar)**

(1) Red
(2) Orange
(3) Yellow
(4) Green
(5) Blue
(6) Violet or Purple
(7) Pink
(8) Brown
(9) Black
(10) White
(11) Grey
(12) Gold
(13) Silver
(99) The brand family name is **not** written in non-Roman characters

B11. Color Descriptors (color_descr_lar)

Are there any color descriptors written on the larger pack (not including brand name)?

A color descriptor is color branding that is used to distinguish multiple packs within a brand. Select any spelling, translation or synonym of the following. Select all that apply, then fill in the text box with the descriptor. Example: a pack with the color descriptor "Ice Blue" would be (5) Blue and then "Ice Blue" entered into the box. In the case of multiple color descriptors, use commas between entries in the text box. Note that flavors are not color descriptors (e.g. "orange mint," "green apple"). This also excludes filter color (e.g. "black filter cigarettes"). Descriptors should be recorded in English.

(1) Red
(2) Orange/Amber
(3) Yellow
(4) Green
(5) Blue
(6) Violet/Purple/Lilac
(7) Pink/Rose
(8) Brown
(9) Black
(10) White
(11) Grey
(12) Gold
(13) Silver
(88) Other
(0) No color descriptors

Selections of 1-13 or 88 prompt text box: **(color_descr_lar_other)**

B12. Tar on Larger Pack (tar_lar)

What is the level of tar on the larger pack (in mg)? If no level listed, enter 99.

B13. Nicotine on Larger Pack (nicotine_lar)

What is the level of nicotine on the larger pack (in mg)? If no level listed, enter 99.

B14. Carbon Monoxide on Larger Pack (carbon_lar)

What is the level of carbon monoxide on the larger pack (in mg)? If no level listed, enter 99.

B15. Prevention of Sale to Youth (youth_lar)

Is there any language on the pack preventing sale to minors? (e.g. "For Adults Only," "Not for Sale to Youth," etc.)

(0) No
(1) Yes

Section C: Primary Package

C1. English on front of the pack (english_front_pri)

Is there any English on the front of the pack, excluding health warnings and brand family name or brand image/crest?

For hard packs, the front of pack is the side where the flip top opens. For soft packs, the front of the pack is the side that is face up when the pack is placed so that the tobacco company text (not the health warning text) on the side of the package is upright and readable. Include words in the url as writing on the front of the pack.

(1) Yes, all of the writing is in English
(2) Yes, some of the writing is in English
(0) No, none of the writing is in English
(99) Not applicable-no other writing except brand family

C2. Type of pack of the primary pack (pack_type_pri)

What is the pack type of the primary package? Choose one.

**Pack Type**	**Description**	**Example**
(1) Hard Pack	Pack with defined shape often constructed out of paper cardboard, which will hold its shape when sticks are removed (could include any shape, e.g. includes lipstick packs).	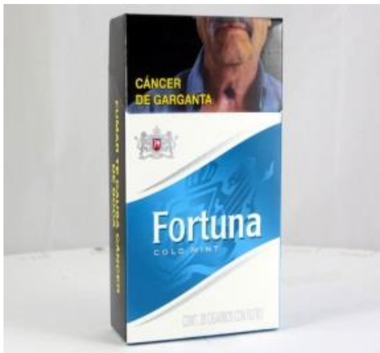
(2) Soft Pack	Pack with malleable shape made of paper or cardboard, with exposed foil or paper.	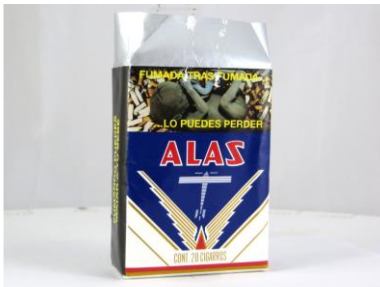
(3) Box	Pack made of tin, other metal or hard plastic.	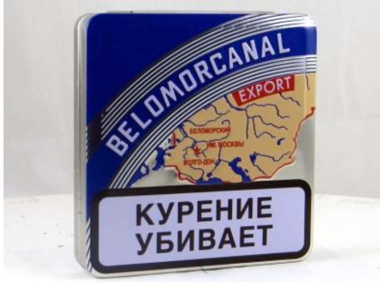
(4) Sachet	Pack made of thin, tear-open plastic, less structured than a soft pack.	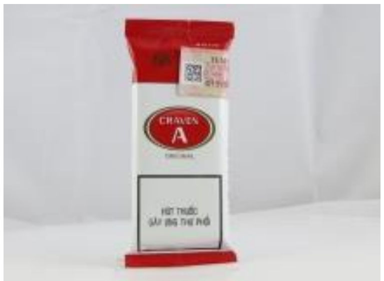
(88) Other	**(pack_type_pri_other)**	

C3. Opening style of primary pack (opening_style_pri)

What is the opening style of the primary pack? Choose one.

**Opening Style**	**Description**	**Example**
(1) Flip-top	Lid is hinged at the back of the pack and when opened reveals the upper portion of the sticks. This excludes flip-tops that open from any other side.	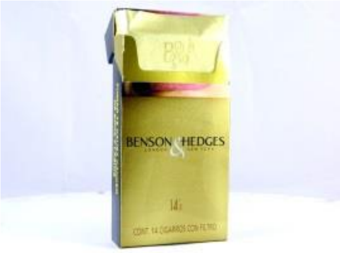
(2) Cigar-box	Lid is hinged at the back of the pack and when opened reveals the full length of the sticks.	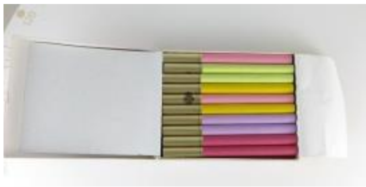
(3) Push-pack	Pack slides open horizontally.	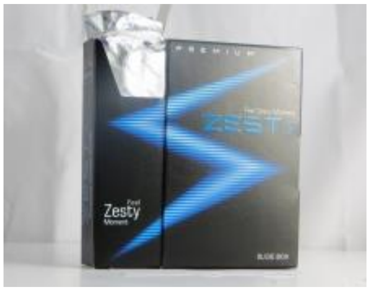
(4) Slide-pack	Pack slides open vertically.	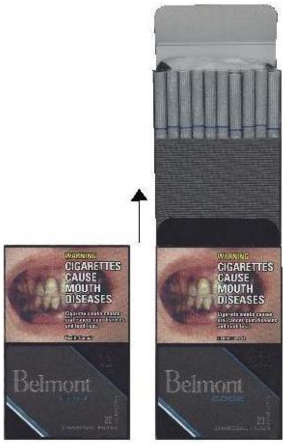
(88) Other	**(opening_style_pri_other)** All other opening styles, including packs that have **both** a flip-top and slide open.	
99) Not Applicable	Not applicable-Pack type is a sachet.	

C4. Shape of the primary pack (pack_shape_pri)

What is the shape of the pack? Choose one.

**Shape**	**Description**	**Example**
(1) Traditional	A rectangular pack with a width to height ratio of approximately 2 to 3; shape resembles a deck of playing cards. This category encompasses packs that may be taller, shorter, smaller or bigger overall, as long as they roughly fit the 2:3 ratio.	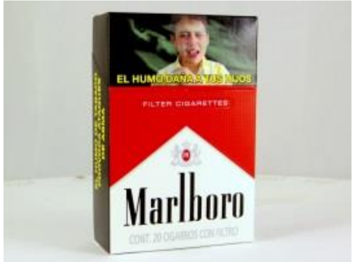
(2) Wide pack	Similar to the traditional pack with wider front and back panel with a width to height ratio greater than 2:3 and less than 1:1.	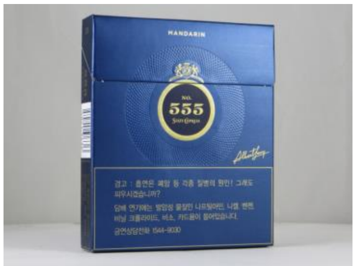
(3) Extra wide pack	Any pack which has a width that is equal to or greater than its height (1:1 or 1>1).	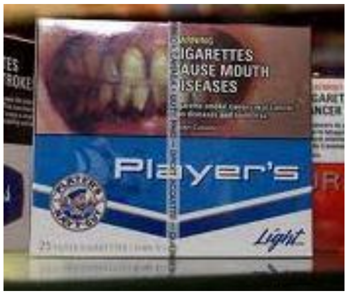
(4) Lipstick pack	A tall, slender pack with roughly equal width for front, back and side panels. Top panel of pack must be square (equal length and width).	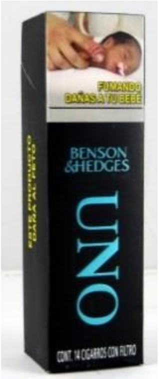
(88) Other	**(pack_shape_pri_other)** All other pack shapes, e.g. packs that appear traditional but unfold further after opening.	
99) Not Applicable	Not applicable-Pack type is a sachet.	

C5. Slimness of pack (slim)

Is the width of the side of the pack 1.3 cm or less?

Measure the entire width using top or bottom of pack in order to avoid measuring on sides with rounded or flattened edges.

(1) Yes
(0) No
(99) Not applicable-Pack type is a sachet

C6. Beveled edges (edges) (skip this question if pack_type_pri is not a Hard Pack)

Does the pack have beveled edges (corners of the pack have been removed)?

**Answer Choice**	**Example**
(1) Yes, the corners of the pack have been flattened.	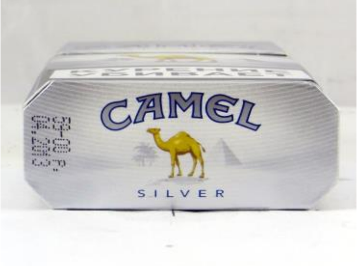
(2) Yes, the corners of the pack are rounded.	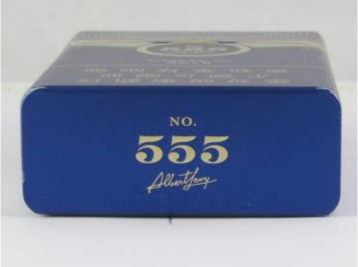
(0) No, the corners are at 90 degree angles.	

C7. Outside fancy features of the primary pack (fancy_finish_pri)

Does the outside of the pack have any of the following features? Select all that apply:

Embossing or debossing can be tested by feeling the features of the outside of the pack.

Holographic printing is any multilayered image that changes content when viewed from different angles.

Iridescent printing refers to any image that changes color when viewed from different angles.

(1) Any embossing or debossing
(2) Shiny or metallic finish that covers the entire front of pack (see front definition)
(3) Shiny metallic detailing
(4) Holographic text, logo or image
(5) Iridescent text, logo or image
(88) Other **(fancy_finish_pri_other)**
(0) No fancy features

C8. Inner foil of pack (inner_foil) (skip this question is pack is soft pack or sachet)

Does the inner foil or paper of the pack have any embellishments? Choose all applicable statements.

**Characteristics of foil or paper:**	**Example**
(0) No, the foil or paper is plain and unembellished (except for instructions like PULL). Silver foil is considered plain and unembellished.	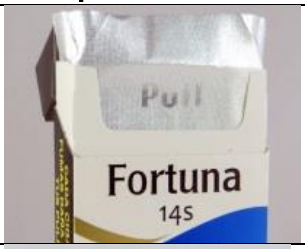
(1) Yes, a color or pattern which is carried through from the outside of the pack (excluding plain silver foil).<br><br>Silver foil never represents a color carried through from the outside of the pack. A pattern generally covers the entirety of the foil and is abstract or consists of a design.	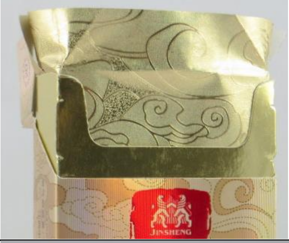
(2) Yes, a color or pattern not otherwise found on the outside of the pack (excluding plain silver foil).	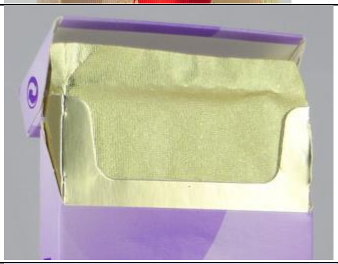
(3) Yes, written words or imagery (not including patterns or PULL).<br><br>This includes words printed in an order or pattern.	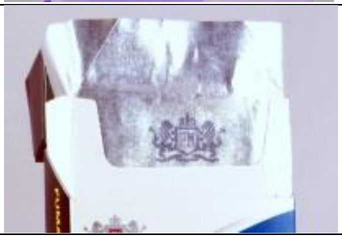
(99) Not Applicable: There is no foil or paper present on the inner part of the pack, pack type is a sachet or soft pack with no foil.	

C9. Additional content under opening (open_content)

When the pack is opened, is there any additional content visible, aside from foil or paper: for example, under the flip-top, under the box lid or other areas that are concealed when the pack is closed? Select all applicable statements.

(1) Contact information for the company (this includes web presence information)
(2) Recycling symbol or terminology
(3) Instructions for how to use stick
(4) Other branding or appeals (e.g. brand name, logo, slogans and designs)
(88) Other **(open_content_other)**
(0) No additional information is visible when the pack is opened
(99) Not applicable-Pack type is a sachet or a soft pack

C10. Change stick flavor (crush_pri)

Does the pack indicate in any way that the user is able to change the stick flavor (e.g. convertibles, click and roll, activate freshness)?

(1) Yes
(0) No

C11. Web Presence (web_pri)

Is there any reference to a web presence on the package? Please select all that apply. This includes both inside and outside of the primary package.

(1) Facebook
(2) Twitter
(3) Company website
(4) Company email
(5) QR code
(88) Other **(web_pri_other)**
(0) No reference to a web presence

C12. Company phone number (phone_pri)

Is a phone number for the company listed anywhere on the primary package? This excludes phone numbers mandated by or associated with health warnings.

(1) Yes
(0) No

C13. Presence of inserts or onserts on primary package (insert_onsert_pri)

Does the primary package contain any inserts or onserts (not including those with only health warning information)? Select all that apply.

An “insert” is any communication inside an individual package and/or carton, such as a miniature leaflet or brochure. An “onsert” means any communication affixed to the outside of an individual package, such as a miniature brochure beneath the outer cellophane wrapping or glued to the outside of the stick package.

(1) Yes, the package contains an insert
(2) Yes, the package contains an onsert
(0) No inserts or onserts

C14. Indication of Stick Size (stick_size_pri)

Describe the size or sizes of the stick listed on the pack. Select all that apply.

For other sizes, enter the English translation. Other stick sizes may include: ultraslims, the stick size written in millimeters, superlarge, etc.

(1) XL or Extra-Large
(2) Kings/King/King Size
(3) Slim/Slims
(4) Superslims
(5) 100s
(6) 120s
(7) Mini, Compact
(88) Other **(stick_size_pri_other)**
(0) No size listed

C15. Colors of Primary Package (colors_pri)

Choose two of the following, looking at the entire pack.

First, select the background color of the primary package, i.e. the dominant color upon which other items are printed.

Next, select a second color. Excluding the background color and the color of the brand name text, what is the one other main prominent color (if any)? If there is no other main color, select "no other main color." The second color should be the other prominent and obvious color at first sight of the pack.

(1) Red
(2) Orange
(3) Yellow
(4) Green
(5) Blue
(6) Violet or Purple
(7) Pink
(8) Brown
(9) Black
(10) White
(11) Grey
(12) Gold
(13) Silver
(0) No other main color

C16. Indication and Color of Brand Family Name – Primary Package

The following questions are designed to capture how the brand family is indicated on the pack and the alphabet and colors in which it is written. The brand family on the pack can be denoted in two different ways: by text/number or by symbol.

A brand family name relates different varieties of products under one umbrella term, number or symbol. Examples: Marlboro, Players, 555. If the brand family is indicated by both text and/or number and symbol and/or image, select text and/or number.

Color questions prompt the list of color options below. If the brand family name is written in two or more colors, choose the color that uses the most space in the name. Iridescent text should be coded for the color of its overall sheen, usually silver. If there are multiple instances of the brand family name, choose the most prominent color.

How is the brand family name indicated on the larger package? **(brandfam_indicate_pri)**

(1) By symbol or image only.
(2) By text and/or number.

A. What is the color of the brand family symbol on the front of the pack? **(color_symbol_pri)**

(1) Red
(2) Orange
(3) Yellow
(4) Green
(5) Blue
(6) Violet or Purple
(7) Pink
(8) Brown
(9) Black
(10) White
(11) Grey
(12) Gold
(13) Silver
(99) Brand family is indicated by text

B. What is the color of the brand family name in Roman characters or Arabic numerals? **(color_roman_text_pri)**

(1) Red
(2) Orange
(3) Yellow
(4) Green
(5) Blue
(6) Violet or Purple
(7) Pink
(8) Brown
(9) Black
(10) White
(11) Grey
(12) Gold
(13) Silver
(99) The brand family name is **not** written in Roman characters or Arabic numerals

C. What is the color of the brand family name in non-Roman characters? **(color_nonroman_text_pri)**

(1) Red
(2) Orange
(3) Yellow
(4) Green
(5) Blue
(6) Violet or Purple
(7) Pink
(8) Brown
(9) Black
(10) White
(11) Grey
(12) Gold
(13) Silver
(99) The brand family name is **not** written in non-Roman characters

C17. Color Descriptors (color_descr_pri)

Are there any color descriptors written on the larger pack (not including brand name)?

A color descriptor is color branding that is used to distinguish multiple packs within a brand. Select any spelling, translation or synonym of the following. Select all that apply. Then fill in the text box with the descriptor. Example: a pack with the color descriptor "Ice Blue" would be (5) Blue and then "Ice Blue" entered into the box. In the case of multiple color descriptors, use commas between entries in the text box. Note that flavors are not color descriptors (e.g. "orange mint," "green apple"). Color descriptors should be recorded in English. This also excludes filter color (e.g. "black filter cigarettes").

(1) Red
(2) Orange/Amber
(3) Yellow
(4) Green
(5) Blue
(6) Violet/Purple/Lilac
(7) Pink/Rose
(8) Brown
(9) Black
(10) White
(11) Grey
(12) Gold
(13) Silver
(88) Other
(0) No color descriptors

Selections of 1-13 or 88 prompt text box: **(color_descr_pri_other)**

C18. Tar on Primary Pack (tar_pri)

What is the level of tar on the primary pack (in mg)? If no level listed, enter 99.

C19. Nicotine on Primary Pack (nicotine_pri)

What is the level of nicotine on the primary pack (in mg)? If no level listed, enter 99.

C20. Carbon Monoxide on Primary Pack (carbon_pri)

What is the level of carbon monoxide on the primary pack (in mg)? If no level listed, enter 99.

C21. Prevention of Sale to Youth (youth_pri)

Is there any language on the pack preventing sale to minors?
(e.g. "For Adults Only," "Not for Sale to Youth," etc.)

(0) No
(1) Yes

Section D: Stick Features

D1. Number of Sticks

A. Count the number of sticks inside the pack. How many sticks are inside of the pack? **(stick_count)**

(1) 20
(2) 14
(3) 10
(4) 5
(88) Other **(stick_count_other)**

B. How many sticks does the pack state it contains? **(stick_state)**

(1) 20
(2) 14
(3) 10
(4) 5
(88) Other **(stick_state_other)**
(99) Not stated on pack

D2. Embellishment on filtertip (filtertip_detail)

If there is a filter on the stick, is the end of the filter embellished in any way; for example with a smiley face, blue dot or recessed filter?

(0) No design
(1) Yes, describe **(filtertip_detail_describe)**
(99) Not applicable–no filter

D3. Filter color (filter_color)

If there is a filter on the stick, what color is the filter?

Includes all shades of the following:

	Example
(1) Cork/Tan	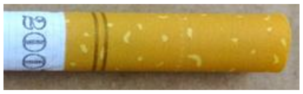
(2) White	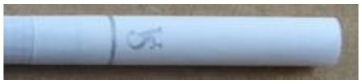
(3) Black	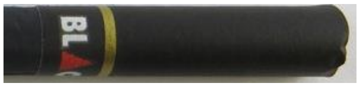
(4) Brown	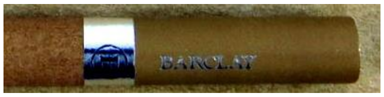
(88) Other color	
(99) No filter	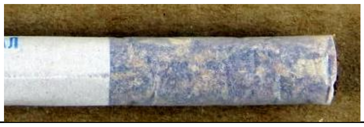

D4. Stick detail (stick_detail)

What branding elements are present on the stick?

Select all that apply. Write all descriptors exactly as they appear on the pack in regards to casing. If the descriptor is in a language other than English, write the English translation of the descriptor. Remove the stick completely from the package and turn the stick to observe all aspects.

**Present on the stick**	**Example**
(1) Brand Family Name (text or number)	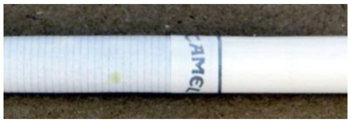
(2) Brand Family Image/Logo/Symbol)<br>*Clarify brand image by checking the brand website. This includes portions of overall brand logos (e.g. crown on stick that is part of a crest on the pack).	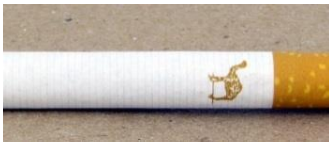
(3) Descriptor or written appeal (e.g,: lights, menthol, red, king size, since 1916) **(brand_descr_describe)**.	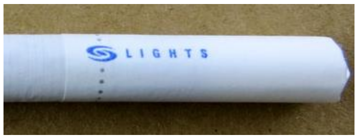
(4) Color carried through from outside of pack. Includes all shades of colors on the outside of the pack.	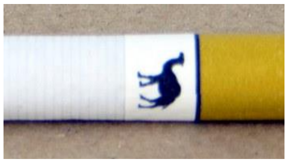 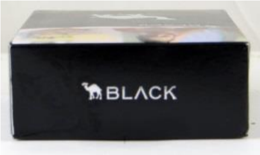
(5) Pattern / design carried through from outside of pack.	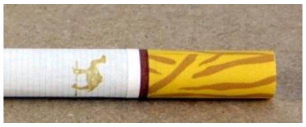 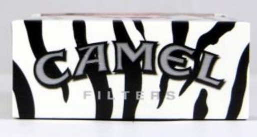
(6) Novel color (not found on outside of pack).	
(7) Novel pattern/design (not found on outside of pack).	
(8) Any symbol for user to "crush" or "turn on" the stick.	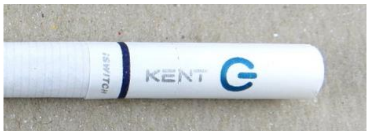
(88) Other: any other imagery branding **(stick_detail_other)**.	
(0) None: stick has no branding.	

D5. Stick Color (stick_color)

What is the color of the stick (the paper after the filter)? Any shade of the following:

(1) White
(2) Black
(3) Brown
(88) Other Color

<u>Section E: Cellophane Wrapping</u> - Refer to pack pictures on TPackSS website for Section E

E1. Cellophane printing (cellophane)

Is there anything printed on the cellophane? Select all that apply.

If there is printing on the cellophane that is illegible, select 0 below and enter the issue into F1.

(1) Yes, a pull tab that is plain and all one color
(2) Yes, a pull tab that has the manufacturer or brand name
(3) Yes, a pull tab that has any kind of branding related to appeals
(4) Yes, any kind of branding related to appeals other than the pull tab
(5) Yes, a pull tab that has health warning labeling
(6) Yes, a pull tab that has duty-free labeling
(0) No, cellophane is plain/translucent or no cellophane

<u>Section F: Topics for Further Discussion</u> - Addressed at coding team review meetings.

F1. Other issues to raise (further_discuss)

Is there any aspect of this pack that needs further discussion?

(1) Yes, describe **(further_describe)**
(0) No

Section G: Appeals

Code the entire pack, including cellophane images, the larger and primary packages, the stick and filter and any items or inserts/onserts that came with the pack, looking for the element of the specific appeal categories described in each question. This includes all appeal words written on the pack, including brand name. This excludes manufacturer, company or factory information and images/logos usually found on the sides of the pack. For example: do not code "British American Tobacco" or the leaves in the British American Tobacco logo.

If any aspect of the pack expresses a particular appeal, select the appropriate item(s) from the list for that aspect. Items in quotations must be found written verbatim. Each element also includes an "other" write-in category. This category is meant to be used for items that fit with the appeals listed here, but are not included on the list and are truly different from the other options.

Words or phrases with quotation marks indicate specificity. For example, do not select the option "technology" unless that exact word is on the pack. Words and phrases without quotation marks signify general themes. For example, it is acceptable to select the option "Since/ Established (year)" if the phrase "for over 100 years" is found on the pack.

A crest should be considered a single item. Do not code imagery or text that is part of a crest.

Which of the following appeal elements appear anywhere on the packaging? Within each appeal, select all elements that apply. If there are no elements of an appeal, select (0) No or (0) None of the above.

G1. Technology Lexical (tech_lex)

(1) Any mention of "technology"
(2) Secondary technology terms (e.g. nano, high-definition, HD, system)
(3) Any term referring to turning off or on (e.g. switch, activate, click, press to refresh)
(4) Words indicating innovation (e.g. new, new generation, innovative)
(5) The phrases "less odor," "odor reducing," "less smoke smell," etc.
(88) Other **(tech_lex_other)** (e.g. anti-counterfeit numbers)
(0) None of the above

G2. Technology Imagery (tech_img)

(1) Power buttons, play buttons, skip track buttons
(2) Any kind of "ball" illustration representing change of flavor (other than buttons)
(3) Stick filter image, if in connection with technology or high-tech filter (e.g. a picture of a stick with a power button on the filter)
(88) Other **(tech_img_other)**
(0) None of the above

G3. Environmental, Civic Responsibility Lexical (environ_lex)

(1) Environmentalism**:** recycle, no littering, "let's protect environment"
(2) Civic Responsibility of the company**:** any program for corporate social responsibility
(88) Other **(environ_lex_other)**
(0) None of the above

G4. Environmental, Civic Responsibility Imagery (environ_img)

(1) Recycle symbol or signs (e.g. green dot symbol, tidy man, three arrows, green leaf)
(88) Other **(environ_img_other)**
(0) None of the above

G5. Organics & Nature Lexical (organic_lex)

(0) Any mention of "natural" or "organic"
(1) Tobacco plant, leaf, leaves (e.g. Broadleaf tobacco, Cuban tobaccos, flue-cured tobacco, etc.)
(2) Nature terminology (e.g. other plants, river, mountain, etc.-Exclude flavors like blueberry, apple, etc.)
(3) Space or star terminology (e.g. celestial bodies like sun, moon; excludes "luxury" stars like "Five Stars")
(88) Other **(organic_lex_other)**
(0) None of the above

G6. Organics & Nature Imagery (organic_img)

(1) Depictions of plants, seedlings, etc. (other than tobacco)
(2) Tobacco plants or leaves
(3) Landscapes of nature scenes (e.g. clouds, rivers, mountains)
(4) Space or star imagery (e.g. celestial bodies like sun, moon; excludes stars used to convey luxury)
(88) Other (**organic_img_other)**
(0) None of the above

G7. Processing of Tobacco Lexical (process_lex)

(1) Blended, blend, mixture
(2) Sun-ripened, slow-roasted, toasted
(3) No added flavor, no additive, pure
(4) Any other description of how tobacco is processed or made into a stick (e.g. dipped, fine-cut)
(88) Other **(process_lex_other)**
(0) None of the above

G8. Luxury and Quality Lexical Elements (lux_lex)

(1) "Quality," "finest" or "fine," "special," "premium"
(2) Class A, #1, Five stars
(3) Gold
(4) References to precious stones (e.g. gems, diamonds)
(5) Traditionally expensive or luxurious items (e.g. silk)
(6) "Export," "Exported," "Import," "Imported"
(7) "Luxury" and all other luxury synonyms (any word or phrase that emphasizes high luxury or quality and is a positive synonym; e.g. superior, perfect, perfection, select, unique, chosen, exceptional, excellence, exclusive, grand, etc.)
(8) Master, professional, expert
(88) Other **(lux_lex_other)**
(0) None of the above

G9. Luxury and Quality Imagery Elements (lux_img)

(1) Crown (if standalone; not part of crest)
(2) Precious stones, gems, diamonds, metals
(3) Traditionally expensive or luxurious items (e.g. fancy cars, rings)
(4) Five-point stars
(88) Other **(lux_img_other)**
(0) None of the above

G10. Classic/Timeless Lexical Elements (classic_lex)

(1) "Classic"
(2) "Original," "Signature"
(3) Traditional
(4) Authentic, genuine, real
(5) Since/Established (year) (includes standalone year)
(88) Other **(classic_lex_other)**
(0) None of the above

G11. Classic/Timeless Imagery Elements (classic_img)

(1) Crest, seal, coat of arms
(2) Castle (if standalone; not part of crest)
(3) Pegasus, Griffin, Lion (if standalone; not part of crest)
(4) Signature (cursive signature of founder)
(88) Other **(classic_img_other)**
(0) None of the above

G12. Femininity Lexical Elements (fem_lex)

(1) Flower terminology (roses, daisies, etc.)
(2) Fashion terms (include style/stylish when paired with other feminine appeals)
(3) Other synonyms for "slim": e.g. slender, skinny, etc. (excludes slim/slims/superslims from B8/C14)
(4) Terms for women, like "Lady" or "Girl"
(88) Other **(fem_lex_other)**
(0) None of the above

G13. Femininity Imagery Elements (fem_img)

(1) Flowers/Butterflies
(2) Fashion imagery
(3) Pastel colors
(4) Non-sexualized female form
(88) Other **(fem_img_other)**
(0) None of the above

G14. Masculinity Lexical Elements (man_lex)

(1) Describe **(man_lex_describe)**
(0) None of the above

G15. Masculinity Imagery Elements (man_img)

(1) Cars
(2) Other vehicles for transportation: planes, ships
(3) Non-sexualized male form
(4) Sports images
(88) Other **(man_img_other)**
(0) None of the above

G16. Youth Lexical (youth_lex)

(1) Describe **(youth_lex_describe)**
(0) None of the above

G17. Youth Imagery (youth_img)

(1) Overall explicit youth appeal on the primary panels (e.g. cartoons, toys/games, imaginary creatures, sports, music)
(0) No youth imagery

Reminder: Exclude company information ("made-in," etc.) for questions G18-G23.

G18. National Lexical (nat_lex)

(1) Mention of any town, city, state or country in which the product was purchased
(2) Famous buildings, structures, or statues
(3) Famous landscapes (e.g. rivers, mountains)
(4) Famous resident (other than leader)
(5) Famous leaders
(6) Country animals (e.g. panda)
(7) Mythical creatures (e.g. dragon)
(8) Country-specific traditional and cultural items identified by expert
(88) Other **(nat_lex_other)**
(0) None of the above

G19. National Imagery (relevant to where product was purchased) (nat_img)

(1) Use of country flag imagery (must have exact imagery and colors from flag)
(2) Famous buildings, structures, or statues
(3) Famous landscapes (e.g. rivers, mountains)
(4) Famous resident (other than leader)
(5) Famous leaders
(6) Country animals (e.g. panda)
(7) Mythical creatures (e.g. dragon)
(8) Country-specific traditional and cultural items identified by expert
(88) Other **(nat_img_other)**
(0) None of the above

G20. Foreign/International (excluding the U.S.) Lexical (foreign_lex)

(1) "International," "world"
(2) Mention of any town, city, state, or country foreign to the country in which the product was purchased
(3) Famous foreign buildings, structures, or statues
(4) Famous foreign landscapes (e.g. rivers, mountains)
(5) Famous foreign resident (other than leader)
(6) Famous foreign leader
(88) Other **(foreign_lex_other)**
(0) None of the above

G21. Foreign/International (excluding the U.S.) Imagery (foreign_img)

(1) Use of foreign flag imagery (must have exact imagery and colors from flag)
(2) Famous foreign buildings, structures, or statues
(3) Famous foreign landscapes (e.g. rivers, mountains)
(4) Famous foreign resident (other than leader)
(5) Famous foreign leader
(88) Other **(foreign_img_other)**
(0) None of the above

G22. United States Lexical (us_lex)

(1) Any mention of America or the U.S.
(2) Any mention of any city, town, or state in the U.S. (excluding Virginia)
(3) Virginia
(4) Famous resident
(88) Other **(us_lex_other)**
(0) None of the above

G23. United States Imagery (us_img)

(1) U.S. flag (must have exact imagery and colors from flag)
(2) U.S. landscape or architecture
(3) Eagle
(4) Idealized/stereotypical Native American
(5) Famous resident
(88) Other **(us_img_other)**
(0) None of the above

G24. Less Harm Lexical Not on Health Warning (less_lex)

(1) Light/Lights
(2) Mild, low
(3) Safe, safer
(4) Soft, smooth, mellow
(5) Any qualitative description of the levels of nicotine, tar or carbon monoxide
(6) Numbers potentially indicating strength. Enter number: **(less_lex_number)**
(88) Other **(less_lex_other)**
(0) None of the above

G25. Less Harm Imagery (less_img)

(1) Image of filter
(2) Dots potentially indicating strength. Enter number of dots: **(less_img_dots)**
(88) Other **(less_img_other)**
(0) None of the above

G26. Less Harm – Filters (less_filter)

Does the pack have any mention of the word "filter," "filters" or "filtered"? Select one.

Filter descriptors must be specifically and blatantly about the filter (e.g. "recessed filter," "triple-action filter"). This excludes descriptors about the cigarette (e.g. "king-size filter cigarettes," "filter-tipped," "black filter cigarettes").

(1) Yes, alone or with the word "cigarettes"
(2) Yes, accompanied by a descriptor about the filter. Enter descriptor: **(filter_descr_describe)**
(0) No

G27. Taste/Sensation Lexical (taste_sens_lex)

(1) Any mention of "taste"
(2) Rich (when referring to taste)
(3) Fresh, freshness, refreshing
(4) Cool, ice, cold, chill, frost
(5) Relax/relaxing
(6) Pleasure, satisfaction, enjoyment
(7) The word "sensation"
(8) Aroma or smell terminology
(88) Other (other taste or sensation descriptors: e.g. balanced) **(taste_sens_other)**
(0) None of the above

G28. Flavor (excludes list of ingredients) Lexical (flavor_lex)

(1) Caramel/vanilla/chocolate
(2) Cinnamon/"canella" or other spice
(3) Clove/Kretek
(4) Menthol
(5) Mint (other than menthol)
(6) Fruit or citrus
(7) Coffee
(8) Alcoholic beverages
(10) Energy drink
(11) Flavor/flavored/flavors
(88) Other **(flavor_lex_other)**
(0) None of the above

G29. Flavor Imagery (flavor_img)

(1) Caramel/vanilla/chocolate
(2) Cinnamon/“canella” or other spice
(3) Clove/Kretek
(4) Menthol
(5) Mint (other than menthol)
(6) Fruit or citrus
(7) Coffee
(8) Alcoholic beverages
(10) Energy drink
(88) Other **(flavor_img_other)**
(0) None of the above

G30. Special Edition Lexical (special_lex)

(1) “Special Edition” or “Limited Edition”
(2) Limited Time: National or community event occurring one time (Olympics, other commemoration)
(3) Limited Time: Holiday (Christmas, Holi, etc.)
(4) Limited Time: Brand event (100 year anniversary)
(88) Other **(special_lex_other)** (excludes color or flavor “editions” such as “red edition,” includes appeals such as “music edition”)
(0) None of the above

G31. Animal Imagery and Terminology (animal)

This selection excludes mythical/imaginary creatures.

(1) Any depiction of an animal (e.g. the camel on Camel cigarettes)
(2) Animal print
(3) Lexical: any mention of an animal
(88) Other **(animal_other)**
(0) None of the above

G32. Other Appeals (add_appeal)

Is there an additional appeal on the pack that does not fit into the categories above?

(1) Yes, describe: **(add_appeal_describe)**
(0) No

G33. Internet Research (research)

Was outside research necessary to clarify an appeal?

(1) Yes, describe: **(research_describe)**
(0) No
